# Distribution of Antibiotic Resistance in a Mixed-Use Watershed and the Impact of Wastewater Treatment Plants on Antibiotic Resistance in Surface Water

**DOI:** 10.3390/antibiotics12111586

**Published:** 2023-11-02

**Authors:** Sohyun Cho, Lari M. Hiott, Quentin D. Read, Julian Damashek, Jason Westrich, Martinique Edwards, Roland F. Seim, Donna A. Glinski, Jacob M. Bateman McDonald, Elizabeth A. Ottesen, Erin K. Lipp, William Matthew Henderson, Charlene R. Jackson, Jonathan G. Frye

**Affiliations:** 1 Poultry Microbiological Safety and Processing Research Unit, Agricultural Research Service, U.S. Department of Agriculture, Athens, GA 30605, USA; sohyun.cho@usda.gov (S.C.); lari.hiott@usda.gov (L.M.H.); charlene.jackson@usda.gov (C.R.J.); 2 Oak Ridge Institute for Science and Education, Oak Ridge, TN 37830, USA; seimroland@unc.edu; 3 Agricultural Research Service, U.S. Department of Agriculture, Southeast Area, Raleigh, NC 27606, USA; quentin.read@usda.gov; 4 Department of Biology, Utica University, Utica, NY 13502, USA; jdamashe@hamilton.edu; 5 Department of Microbiology, University of Georgia, Athens, GA 30602, USA; jwestrich@rwdc-industries.com (J.W.); ottesen@uga.edu (E.A.O.); 6 Department of Environmental Health Science, University of Georgia, Athens, GA 30602, USA; mle9@iu.edu (M.E.); elipp@uga.edu (E.K.L.); 7 Center for Environmental Measurement and Modeling, Office of Research and Development, U.S. Environmental Protection Agency, Athens, GA 30605, USA; glinski.donna@epa.gov (D.A.G.); henderson.matt@epa.gov (W.M.H.); 8 Lewis F. Rogers Institute for Environmental and Spatial Analysis, University of North Georgia, Oakwood, GA 30566, USA; jacob.batemanmcdonald@ung.edu

**Keywords:** antibiotic-resistant bacteria, antibiotic resistance gene, antibiotic, freshwater, environment, wastewater

## Abstract

The aquatic environment has been recognized as a source of antibiotic resistance (AR) that factors into the One Health approach to combat AR. To provide much needed data on AR in the environment, a comprehensive survey of antibiotic-resistant bacteria (ARB), antibiotic resistance genes (ARGs), and antibiotic residues was conducted in a mixed-use watershed and wastewater treatment plants (WWTPs) within the watershed to evaluate these contaminants in surface water. A culture-based approach was used to determine prevalence and diversity of ARB in surface water. Low levels of AR *Salmonella* (9.6%) and *Escherichia coli* (6.5%) were detected, while all *Enterococcus* were resistant to at least one tested antibiotic. Fewer than 20% of extended-spectrum β-lactamase (ESBL)-producing *Enterobacteriaceae* (17.3%) and carbapenem-resistant *Enterobacteriaceae* (CRE) (7.7%) were recovered. Six ARGs were detected using qPCR, primarily the erythromycin-resistance gene, *erm*B. Of the 26 antibiotics measured, almost all water samples (98.7%) had detectable levels of antibiotics. Analysis of wastewater samples from three WWTPs showed that WWTPs did not completely remove AR contaminants. ARGs and antibiotics were detected in all the WWTP effluent discharges, indicating that WWTPs are the source of AR contaminants in receiving water. However, no significant difference in ARGs and antibiotics between the upstream and downstream water suggests that there are other sources of AR contamination. The widespread occurrence and abundance of medically important antibiotics, bacteria resistant to antibiotics used for human and veterinary purposes, and the genes associated with resistance to these antibiotics, may potentially pose risks to the local populations exposed to these water sources.

## 1. Introduction

There is a growing recognition of aquatic environments as a reservoir and transmission route through which antibiotic resistance (AR) can be spread [1,2,3,4]. Surface water constantly receives pathogenic and non-pathogenic bacteria from diverse sources including wastewater treatment plants (WWTPs), septic systems, wildlife, and agriculture. Bacteria released into the environment may be resistant to antibiotics, acting as reservoirs of antibiotic resistance genes (ARGs) that can be transferred to environmental bacteria and/or pathogenic bacteria present in the same niche through horizontal gene transfer [5,6].

Antibiotic-resistant bacteria (ARB) in the aquatic environment may pose a risk to human health, as they can be ingested by humans and animals through drinking water, recreational activities, and consumption of produce irrigated with contaminated water. In particular, the emergence and spread of extended-spectrum β-lactamases (ESBLs) and carbapenem-hydrolyzing β-lactamases (i.e., carbapenemases) in *Enterobacteriaceae* are a clinical concern as they confer resistance to most β-lactam antibiotics [7]. They are also often characterized by multidrug resistance (MDR) against drugs commonly used to treat Gram-negative bacterial infections, leaving limited therapeutic choices [8,9,10]. ESBL and carbapenemase genes are primarily located on mobile genetic elements, such as plasmids, and can easily spread to bacteria present in the environment [9,10]. In the light of the One Health approach, it is important to understand the abundance and distribution of ARB and ARGs in the environment, as their presence in surface waters is a public health concern.

The emergence of ARB populations in surface water may be favored by the presence of antibiotic residues discharged into the receiving water along with agricultural, municipal, and industrial wastewater [2]. Substantial proportions of antibiotics consumed by humans and animals, which are excreted via urine and feces in their active forms, and unused antibiotics inappropriately disposed of by flushing down toilets enter the waste stream [11]. However, since WWTPs are not constructed to remove antibiotics, substantial volumes of antibiotics are discharged into the environment [11,12,13]. For example, a study conducted in China documented that 58% of the antibiotics consumed by humans and animals entered terrestrial (i.e., sludge application) and aquatic (i.e., wastewater discharge) systems through wastewater treatment [13]. The presence of antibiotics in the environment has a deleterious effect since low concentrations of antibiotics, even below their minimal inhibitory concentrations (MICs: the lowest antibiotic concentrations above which bacterial growth is prevented), have been shown to select for AR phenotypes [14,15,16] and also cause AR development by inducing horizontal gene transfer [17,18] and mutagenesis [19,20].

In this study, the abundance and distribution of ARB and ARGs, as well as the concentrations of antibiotics present in a mixed-use watershed in Athens, GA, USA were examined, in order to enhance understanding of the existing state of AR in the freshwater environment. The Upper Oconee Watershed is a mixed-use watershed that covers agricultural, forested, industrial, and residential areas. It is comparable to other similar-sized watersheds that contain both agricultural and urban areas and are impacted by contamination from human and animal sources. Since watersheds are frequently used for recreational purposes and serve as the source of drinking water to local municipal areas, it is important to assess the extent of AR contamination in surface water and determine the potential sources of the contamination. ARB from surface water were isolated to characterize the bacteria that express ARGs, and the total ARGs present within the watershed were quantified, including genes that were not expressed. The distribution and concentration of antibiotics that are important to human and veterinary medicine were also estimated. This study was designed to include sites impacted by agricultural and anthropogenic pollution sources. As WWTPs are considered hotspots for the development of ARB and the source of their spread into the environment [21], untreated influent and treated effluent from the three WWTPs located within the study area were included in the analyses. These WWTPs serve residences, industrial facilities, hospital facilities, and a university campus within the watershed areas and discharge treated wastewater into the streams within the watershed. The influent and effluent samples from the WWTPs were compared to investigate WWTPs as a potential source of AR contaminants in the natural environment.

## 2. Results

### 2.1. Prevalence and Distribution of AR Contaminants in Surface Water

#### 2.1.1. Antibiotic-Resistant *Salmonella*, *Escherichia coli*, and *Enterococcus*

*Salmonella* was detected in 68.8% (117/170) of water samples, with the highest detection rate observed in summer (93.2%; 41/44) and the lowest detection rate observed in winter (48.8%; 20/41) (Table 1). One-tenth of *Salmonella* isolates were resistant to at least one antibiotic (9.6%; 29/303), while 27 isolates exhibited MDR (resistance to three or more antibiotic classes) phenotypes with resistance to 10 antibiotics, including ceftiofur and ceftriaxone, which are third-generation cephalosporin drugs. These MDR isolates were all recovered during the fall collection from 10 different sampling sites along four flow-paths, including five sites along McNutt Creek (Supplementary Materials Table S1). The detection rates of *Salmonella* in wastewater influent and effluent were 100% (12/12) and 9.1% (1/11), respectively, and the AR rate was 24% (6/25) of the total *Salmonella* isolates, with all the resistant *Salmonella* recovered from influents.

Nearly all surface water and wastewater samples were positive for *E. coli* and *Enterococcus* (Table 1). However, only 8.4% (16/191) of the total *E. coli* isolates from surface water and wastewater exhibited resistance to antibiotics tested. This corresponds to the AR rates of 6.5% (11/170) and 23.8% (5/21) for *E. coli* in surface water and wastewater, respectively. Resistance to all the 14 drugs tested was observed in *E. coli* isolates, up to seven antibiotics per isolate, and resistance was observed most frequently to tetracycline (n = 7) and ampicillin (n = 4) (Tables S1–S4). Simultaneously, a total of 169 and 22 *Enterococcus* isolates were recovered from surface water and wastewater, respectively, and all the isolates were resistant to at least one of the 16 drugs tested (Table 1). Eight isolates from surface water (4.7%; 8/169) and five isolates from wastewater (22.7%; 5/22) exhibited MDR phenotypes. The most common resistance in *Enterococcus* was against lincomycin (98.8%; 167/169), followed by tetracycline (13.0%; 22/169) (Tables S1–S4).

#### 2.1.2. ESBL-Producing *Enterobacteriaceae* and Carbapenem-Resistant *Enterobacteriaceae* (CRE)

Selective isolation on antibiotic-containing media yielded a total of 455 bacterial isolates, which were then screened by PCR for 11 β-lactamase genes. This resulted in 147 β-lactamase-positive *Enterobacteriaceae* isolates (excluding duplicate organisms isolated from the same site on the same collection date with the same AR phenotypes and ARG patterns) from 48.8% (82/168) of surface water samples and 70.8% (17/24) of wastewater samples. The β-lactamase-positive *Enterobacteriaceae* isolates included *Citrobacter braakii*/*freundii* (n = 1), *C. freundii* (n = 1), *Enterobacter asburiae* (n = 2), *Enterobacter cloacae* complex (n = 10), *Enterobacter* spp. (n = 2), *E. coli* (n = 18), *Klebsiella oxytoca* (n = 1), *Klebsiella pneumoniae* (n = 11), *Kluyvera ascorbata* (n = 1), *Kluyvera cryocrescens* (n = 3), and *Serratia fonticola* (n = 97) (Table 2).

Almost all the β-lactamase-positive *Enterobacteriaceae* were phenotypically resistant to at least one of the 16 antibiotics tested (99.3%; 146/147), and 143 (97.3%) isolates were resistant to two or more antibiotics (Tables S1–S4). A total of 88 (59.9%) isolates were resistant to antibiotics that belong to higher generations (third or fourth) of cephalosporins and/or carbapenems. The remaining 59 isolates were resistant to other β-lactams and first and second generations of cephalosporins, most of which were *S. fonticola* (n = 56). Of the 147 β-lactamase-positive *Enterobacteriaceae* isolates, 42 were phenotypically confirmed as inhibitor-sensitive ESBL-producers and 18 were phenotypically confirmed as CRE, out of which two *S. fonticola* isolates exhibited both the phenotypes (Table 2). The recovery rates of ESBL-producing *Enterobacteriaceae* and CRE isolates were 17.3% (29/168) and 7.7% (13/168) for surface water samples, respectively. On the other hand, ESBL-producing *Enterobacteriaceae* and CRE isolates were detected in half of wastewater samples (50%; 12/24), with 58.3% (7/12) and 41.7% (5/12) recovery rates for influents and effluents, respectively.

Among ESBL producers and CRE, *bla*_CTX-M_ was the most frequently detected gene (n = 43), followed by *bla*_SHV_ (n = 9), *bla*_IMI-NmcA_ (n = 8), *bla*_TEM_ (n = 8), *bla*_KPC_ (n = 5), *bla*_OXA-1_ (n = 3), and *bla*_CMY-2_ (n = 3) (Table S5). All *bla*_CTX-M_-carrying *E. coli* (n = 14) exhibited the ESBL-producing phenotype, with three isolates carrying an additional plasmid-mediated AmpC β-lactamase gene, *bla*_CMY-2_. ESBL-producing bacteria and β-lactamase-containing bacteria were less likely to be present in fall compared with the other seasons (Table S6).

#### 2.1.3. Antibiotic Resistance Gene Markers

Using qPCR, ARGs were detected in 67.2% (39/58) of the water sites that were sampled at least once during the collection periods. The most frequently detected resistance gene was *erm*B, which was present in 33.3% (50/150) of the total surface water samples, followed by *qnr*S (18.2%; 27/148), *bla*_KPC_ (15.4%; 23/149), *tet*B (10.1%; 15/148), *bla*_SHV_ (9.4%; 14/149), and *bla*_CTX-M_ (2.0%; 3/148) (Table 3). Fall was the season when ARGs were detected the most, with 63.2% (24/38) of the sampling sites positive for ARGs, including NORO 520 and NORO 615 where all six genes were detected (Tables S1–S4). This was followed by summer, winter, and spring, when 35.0% (14/40), 31.6% (12/38), and 23.5% (8/34) of the sites were positive for ARGs, respectively. Except for *erm*B during fall, when the detection rate in surface water was 60.5%, all the other genes had lower rates of detection throughout the year below 30%. All wastewater influent samples were positive for every ARG for all seasons analyzed (Table 3).

North Oconee River, including the aforementioned NORO water sampling sites, was the most polluted water in terms of ARG abundance. The highest copy numbers of *erm*B (1533.8 copies/mL in NORO 520 in fall), *tet*B (127.0 copies/mL in NORO 520 in fall), *bla*_KPC_ (377.5 copies/mL in NORO 108 in summer), and *qnr*S (703.0 copies/mL in NORO 108 in summer) were detected from the sampling sites along the North Oconee River (Table 3, Tables S1 and S4).

#### 2.1.4. Antibiotics

All 26 antibiotics were detected in surface water, and all 151 samples across 57 sites had detectable levels of antibiotics except two samples in winter. In surface water, trimethoprim was the most frequently detected antibiotic (89.4%; 135/151), followed by tylosin (60.3%; 91/151) and sulfamethoxazole (48.3%; 73/151), while the least detected antibiotics were kanamycin (3.3%; 5/151), tigecycline (3.3%; 5/151), and vancomycin (6%; 9/151) (Table 4). The concentrations of the measured antibiotics in surface water generally varied by season during the sampling year. Spring was the season with the highest average antibiotic concentrations, whereas fall exhibited the lowest levels of antibiotics with an approximately 6.8-fold higher concentration in spring compared with fall (OR = 0.148, 95% CI [0.0464, 0.467]). Daptomycin, ceftriaxone, and oxacillin were found with the highest average concentrations, but it was because their antibiotic concentrations were highly elevated in one seasonal collection, rather than maintaining high concentrations throughout the year. There were seasons when the maximum levels of antibiotics at certain sites were particularly high, with more than a 10-fold increase compared with the average concentrations; approximately 100-fold increases were also observed, such as in daptomycin during winter (1472.8 ng/L vs. annual average of 15.5 ng/L) and ceftriaxone during spring (750.5 ng/L vs. annual average of 12.7 ng/L).

**Figure 1:**
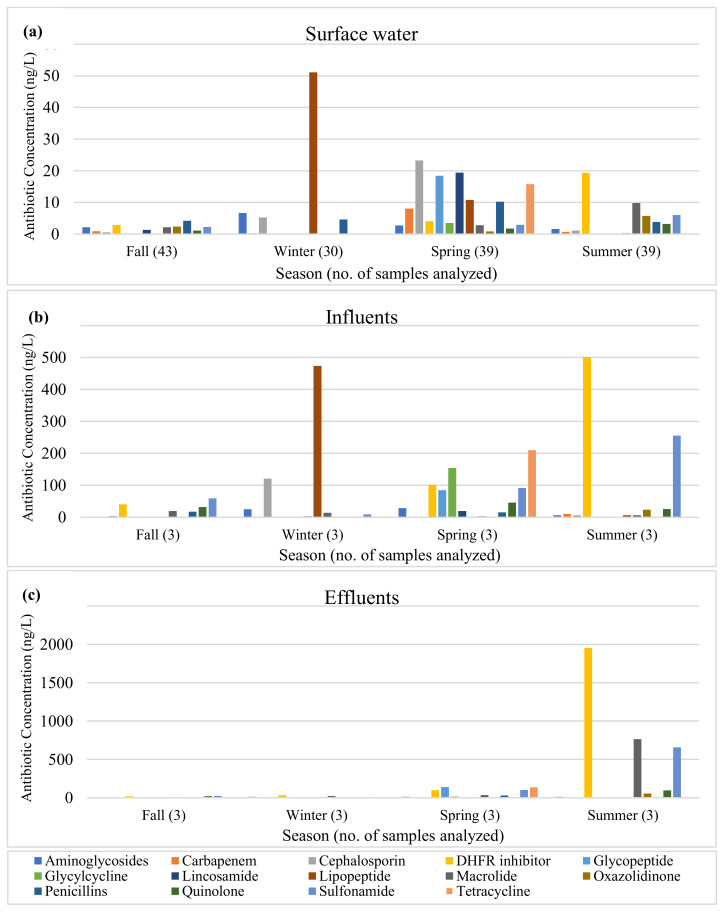
Antibiotic concentrations in (**a**) surface water, (**b**) influents, and (**c**) effluents by antibiotic class.

While all 26 antibiotics were detected in wastewater influent samples, ceftriaxone was not detected in effluent samples (Tables S1–S4). Sulfamethoxazole was the most frequently detected antibiotic in wastewater, detected in all 24 wastewater samples, followed by trimethoprim (91.7%; 22/24), tylosin (75.0%; 18/24), and azithromycin (70.8%; 17/24), while ceftriaxone was the least detected antibiotic (4.2%; 1/24) (Table 4). In terms of antibiotic concentrations, trimethoprim had the highest concentration in wastewater samples, followed by sulfamethoxazole and erythromycin, with average concentrations of 343.3 ng/L, 292.7 ng/L, and 262.4 ng/L, respectively (Table 4). The results of this study showed that most antibiotics were still present in WWTP effluents at high concentrations. By class, DHFR inhibitors, sulfonamides, and cephalosporins were present in highest levels in influents, while DHFR inhibitors, sulfonamides, and macrolides were present in highest levels in effluents (Figure 1). During summer, DHFR inhibitors, macrolides, and sulfonamides were present at much higher concentrations in effluents than in influents.

### 2.2. WWTPs as a Source of AR Contaminants

**Figure 2:**
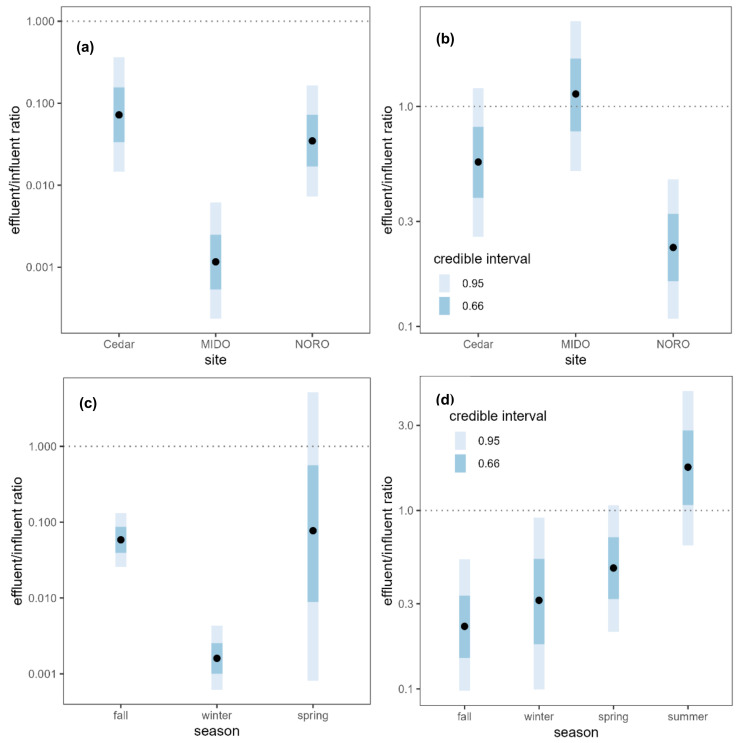
Absolute ARG copy number ratio between wastewater influents and effluents (effluent:influent) (**a**) by site and (**c**) by season, and antibiotic concentration ratio between wastewater influents and effluents (effluent:influent) (**b**) by site and (**d**) by season. Black points represent the median of the posterior distributions of the effluent:influent ratios. Dotted lines are plotted at a ratio of 1:1 indicating no difference between influent and effluent.

**Figure 3:**
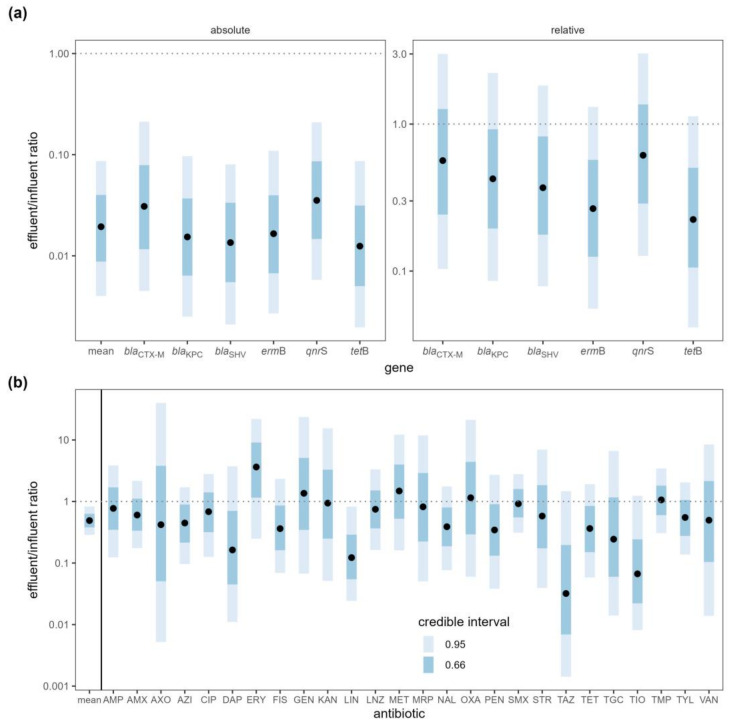
(**a**) ARG copy number ratio and (**b**) antibiotic concentration ratio between wastewater influent and effluent samples (effluent:influent). Antibiotics: amoxicillin (AMX), ampicillin (AMP), azithromycin (AZI), ceftriaxone (AXO), ceftazidime (TAZ), ceftiofur (TIO), ciprofloxacin (CIP), daptomycin (DAP), erythromycin (ERY), gentamicin (GEN), kanamycin (KAN), lincomycin (LIN), linezolid (LNZ), meropenem (MRP), methicillin (MET), nalidixic acid (NAL), oxacillin (OXA), penicillin (PEN), sulfamethoxazole (SMX), sulfisoxazole (FIS), streptomycin (STR), tigecycline (TGC), trimethoprim (TMP), tetracycline (TET), tylosin (TYL), and vancomycin (VAN). Black points represent the median of the posterior distributions of the effluent:influent ratios. Dotted lines are plotted at a ratio of 1:1 indicating no difference between influent and effluent.

After wastewater treatment, the absolute copy number of each ARG was reduced to 1% to 3% of its original quantity, with the average reduction to 2% of the original quantity (effluent:influent ratio = 0.0194, 95% CI [0.00401, 0.0865]) (Table S7). The removal efficiency of ARGs was compared among the WWTPs, and although all WWTPs were effective at reducing ARG concentrations, the MIDO WWTP exhibited the highest removal efficiency (Figure 2a). The Cedar (OR = 0.07210, 95% CI [0.0147, 0.365]) and NORO (OR = 0.03470, 95% CI [0.00726, 0.165]) WWTPs reduced the ARG concentrations to approximately 5% of the influents, while the MIDO WWTP reduced it to approximately 0.1% of the influent (OR = 0.00116, 95% CI [0.000235, 0.00617]), confirming that it was most effective in removing ARGs. Although wastewater treatment greatly reduced the absolute copy numbers of ARGs, the relative copy numbers (normalized to 16S rRNA gene copy numbers) were not significantly reduced (Figure 3a).

WWTPs did not remove antibiotics as efficiently as ARGs. When antibiotic concentrations in the influent and effluent samples were compared, the mean antibiotic concentration decreased by half (ratio = 0.49, 95% CI [0.287, 0.83]) upon treatment (Figure 3b, Table S8). This difference was driven by small decreases in most of the antibiotics, although the majority of these decreases were not statistically significant on an individual basis. The concentrations of 21 antibiotics decreased while the concentrations increased for five antibiotics (erythromycin, gentamicin, methicillin, oxacillin, trimethoprim), but none of the changes were statistically meaningful except for lincomycin (ratio = 0.1220, 95% CI [0.0243, 0.823]). The NORO WWTP was most effective in removing antibiotics while the other two WWTPs were not effective in reducing antibiotic concentrations (Figure 2b). The NORO WWTP reduced the concentration by a factor of four from influent to effluent (OR = 0.228, 95% CI [0.108, 0.467]), while the MIDO WWTP did not remove antibiotics at all (OR = 1.140, 95% CI [0.509, 2.44]).

Seasonal variability in the efficiency of the wastewater treatment was examined. The removal efficiency of ARGs was highest in winter (Figure 2c), when almost all ARGs were removed with less than 1% of the copy numbers remaining after the treatment (ratio = 0.0016, 95% CI [0.000617, 0.00432]). In contrast, there was no evidence for reduction in the absolute ARG copy numbers during fall and spring, although only the comparison between winter and fall showed a significant difference (ratio of removal efficiencies = 36.5, 95% CI [10.2, 127]). Summer was excluded from the analysis as no wastewater samples were available for the ARG analysis. On the other hand, the removal efficiency of antibiotics was highest in fall and lowest in summer (Figure 2d). Wastewater treatment was not effective at all in summer when the incoming concentration of antibiotics was highest. The effectiveness of wastewater treatment in reducing antibiotic concentration was greater in fall (ratio = 7.813, 95% CI [2.381, 25.773]), winter (ratio = 5.61, 95% CI [1.27, 24.5]), and spring (ratio = 3.65, 95% CI [1.071, 12.092]) relative to summer.

Water samples collected upstream (n = 20) and downstream (n = 4) of the NORO WWTP were compared to evaluate the impact of treated effluent on surface waters. A relatively higher concentration of ARGs and a lower concentration of antibiotics were detected in the downstream water compared with the upstream water, although the differences were not statistically meaningful (Table S9).

## 3. Discussion

### 3.1. Prevalence and Distribution of AR Contaminants in Surface Water

#### 3.1.1. Antibiotic-Resistant *Salmonella*, *E. coli*, and *Enterococcus*

Culture-based methods were used to investigate AR in *Salmonella*, *E. coli*, and *Enterococcus* present in the freshwater environment. Chromogenic media without any antibiotic supplements were used to isolate the bacteria, and their resistance to antibiotics was determined using susceptibility testing to estimate the level of AR within the bacterial community in the aquatic environment. *Salmonella*, *E. coli*, and *Enterococcus* were chosen for the study as these bacteria are often used as sentinel organisms for monitoring the overall trends in resistance to antibiotics in Gram-negative and Gram-positive bacteria [22].

This study has shown that the majority of *Salmonella* and *E. coli* from surface water and wastewater were susceptible to the antibiotics tested. The 27 MDR *Salmonella* recovered from surface water during the fall collection were found to be *S*. Oranienburg clones [23]. Considering that the wide occurrence of MDR *S*. Oranienburg was an unusual observation that may be due to a specific event from a common source of contamination, the occurrence of AR *Salmonella* seems to be even more uncommon in the Upper Oconee Watershed. Apart from these MDR *S*. Oranienburg isolates, only two resistant *Salmonella* were detected from surface water. Resistance to a relatively new antibiotic, daptomycin, was detected, mostly in *Enterococcus hirae*, similar to the previous studies conducted in the same watershed; however, resistance to another newer drug, tigecycline, was not observed during this sampling period [24,25].

#### 3.1.2. ESBL-Producing *Enterobacteriaceae* and CRE

For the detection of ESBL-producing *Enterobacteriaceae* and CRE, selective media supplemented with antibiotics were used, as the presence of these bacteria in the aquatic environment was expected to be very low. In our previous study, only one ESBL-producing *E. coli* was recovered from the same watershed out of 496 *E. coli* isolates when culture media without antibiotic supplements was used [26].

More than half of the isolates positive for β-lactamase genes were *S. fonticola*, all of which carried *bla*_CTX-M_. It has been shown that *S. fonticola* possess a chromosomally encoded ESBL *bla*_FONA_ gene which is closely related to the *bla*_CTX-M_ gene and thus can be amplified by the CTX-M-type β-lactamase gene PCR assay [27,28]. Since *bla*_FONA_ was not one of the target genes for this study, a further analysis to verify the gene was not conducted, but it is very probable that the β-lactamase gene found in our *S. fonticola* isolates was actually an intrinsic *bla*_FONA_ gene. Interestingly, all of *S. fonticola* isolates were from surface water, indicating the environmental origin of *S. fonticola* and that they are widely disseminated in the aquatic ecosystem. Previous studies also suggest an environmental origin for *S. fonticola*, as β-lactamase-positive *S. fonticola* have been frequently isolated from vegetables, birds, soil, and surface water [29,30,31,32] and a carbapenem-resistant *S. fonticola* was isolated from drinking water [33]. Although *S. fonticola* is not typically pathogenic, the presence of ESBL-producing and carbapenem-resistant *S. fonticola* in the environment could be a public health concern as they could be vectors for the AR dissemination in the environment and to humans. It is possible for the chromosomally-encoded *bla*_FONA_ gene to be mobilized on a mobile genetic element and transferred to pathogenic bacteria, just as chromosomally encoded β-lactamase genes in *Kluyvera* spp. are thought to have been mobilized to become the precursor genes for plasmid-encoded CTX-M-type ESBL genes [34,35].

Ten isolates identified as *E. cloacae* complex and two isolates of *E. asburiae*, which is a species within the *E. cloacae* complex, were detected with either *bla*_KPC_ or *bla*_NMC-A/IMI_ (not sequenced to discriminate between NMC-A and IMI enzymes). Of these 12 isolates, 10 isolates were carbapenem resistant, positive for either *bla*_KPC_ (n = 2) or *bla*_NMC-A/IMI_ (n = 8). In the US, the prevalence of carbapenem-resistant *K. pneumoniae* carrying *bla*_KPC_ is declining, while an increase in the prevalence of carbapenem-resistant *E. cloacae* complex has been reported [36,37]. *E. cloacae* most commonly acquire carbapenem resistance through plasmid-encoded carbapenemase genes, such as *bla*_KPC_ and *bla*_NDM_, or through overexpression of AmpC [37]. However, *E. cloacae* complex with chromosomally-encoded β-lactamase genes *bla*_NMC-A_ and *bla*_IMI_ has been occasionally reported [38,39,40]. Our current study suggests that NMC-A/IMI-producing *E. cloacae* complex may be more common in the environment than it has been reported. Interestingly, all carbapenem-resistant *E. cloacae* complex, except one isolate identified as *E. asburiae*, were recovered during the summer collection from either wastewater effluent or surface water, one of which was downstream of a WWTP. This shows a possible seasonal prevalence of carbapenem-resistant *E. cloacae* complex in surface water, as well as a possible release of these organisms from WWTPs to surface water as they escape the wastewater treatment processes. Recently, *bla*_NMC-A/IMI_ genes were found to be harbored on integrative mobile elements within the host chromosome or plasmids to enhance their potential to be mobilized [39,41,42]. Although the location of *bla*_NMC-A/IMI_ was not determined in this study, it is possible that this carbapenemase gene can be mobilized and transferred to other bacteria.

The lowest number of β-lactamase-positive *Enterobacteriaceae* was recovered in fall, mainly because *S. fonticola*, the most prevalent organism with β-lactamase genes, was not recovered during this season. The absence of *S. fonticola* in fall could be due to a seasonal difference but could also be due to a modification in the isolation method. An exclusion of sample enrichment and usage of different selective media in fall could be responsible for the recovery of different bacterial composition as the media components were previously shown to affect the composition of bacteria recovered [24,43].

Some of the AR bacteria isolated in surface water and wastewater were the ESKAPE pathogens (*Enterococcus faecium*, *Staphylococcus aureus*, *Klebsiella pneumoniae*, *Acinetobacter baumannii*, *Pseudomonas aeruginosa*, and *Enterobacter* spp.) which are the leading cause of nosocomial infections [44]. Their multidrug resistance phenotypes and the clinical and economic burdens of the infections have made them the AR “priority pathogens” by WHO since they represent a global threat to human health [45]. MDR ESKAPE pathogens, including *E. faecium*, *K. pneumoniae*, and *Enterobacter* spp. were detected in our watershed, some of which were ESBL and carbapenemase producers, showing that these pathogens are present outside the clinical environment in the natural environment.

#### 3.1.3. Antibiotic Resistance Gene Markers

Six ARGs, namely *erm*B, *tet*B, *qnr*S, *bla*_KPC_, *bla*_SHV_, and *bla*_CTX-M_, were chosen as they were reported to be commonly detected in the same watershed and represent resistance to a wide range of antibiotics [46]. As reported in Damashek et al., the presence of these six ARGs was investigated and their copy numbers were quantified using qPCR [46]. While the ARG copy numbers ranged from 10^3^ to 10^5^ copies/mL in wastewater influents and 10^0^ to 10^5^ copies/mL in wastewater effluents, the copy numbers ranged from 10^0^ to 10^3^ copies/mL in surface water. The copy numbers of ARGs in wastewater were reduced during the treatment process (discussed below) which could have been further reduced in surface water due to a dilution effect. The rate of detection of the β-lactam resistance gene *bla*_KPC_ was higher than those of *bla*_SHV_ and *bla*_CTX-M_, while *bla*_CTX-M_ was the most recovered gene using the culture-based method. This shows that the frequency of β-lactamase gene detection may vary using different detection methods. The culture-independent method would measure ARGs within bacterial populations that cannot be cultured, which may have contributed to the difference in the ARG detection rates.

#### 3.1.4. Antibiotics

A liquid chromatography tandem mass spectrometry (LC-MS/MS) method was used to detect and quantify 26 antibiotics representing 14 different classes, and the results indicate the widespread presence of antibiotic residues across the watershed. Trimethoprim and sulfisoxazole are used in combination to treat a wide variety of bacterial infections, which may have increased the detection frequency of both the antibiotics in surface water and wastewater. The prevalence of trimethoprim was higher than sulfamethoxazole most likely because trimethoprim is also used with other sulfonamides. Penicillins, macrolides, and cephalosporins were the top oral antibiotic classes prescribed in the US in the years 2017 and 2018, during which this study was undertaken, reflecting their extensive usage and thus their prevalence in the environment [47,48]. Tylosin, typically used to treat infections in farm animals and companion animals, is a widely used antibiotic that was applied as a growth promoter until the ban of the practice in the US in January 2017, after which it remained in continuous use for disease prevention in animals [49]. Its common usage in animals may have increased its presence in the environment. Despite its frequent prescription in the US, tetracycline was only detected a few times and at low concentrations in this study [48]. This could be due to rapid degradation of tetracycline upon light exposure or binding of tetracycline to suspended matter, which makes the antibiotic difficult to be detected as free molecules [50,51]. Daptomycin, a lipopeptide antibiotic, was present at a very high concentration in the influent samples in winter, suggesting its high usage during winter season, but the wastewater treatment process was able to reduce it to a low concentration. Nevertheless, the concentration of daptomycin in water samples was high, suggesting that WWTPs may not be the major source of antibiotic pollution in surface water for some antibiotics. The high concentrations of daptomycin found in surface water may have contributed to daptomycin resistance in the environmental isolates, while daptomycin resistance is not very common in clinical isolates [24,25].

During fall and summer, most of the water sites did not exceed antibiotic concentrations of 100 ng/L, an antibiotic discharge limit for protecting bacterial populations in the environment and preventing the risk of AR development [52]. In contrast, antibiotic concentrations often exceeded 100 ng/L during winter and spring, especially during spring when the concentrations in surface water were often higher than those found in wastewater. Seasonality of antibiotic residues present in the environment was expected to be driven by the seasonality of antibiotic use. Higher levels of antibiotic residues were predicted in winter as more antibiotics are prescribed in the winter months with the increased incidence of infections, especially respiratory tract infections [53]. However, spring was the season with the highest concentrations of antibiotics in surface water. Athens, GA, includes a university campus with higher student populations during fall and spring seasons, which may have increased the use of antibiotics, thus resulting in higher concentrations of antibiotics in the environment in spring. Seasonality could also be associated with the chemical characteristics of antibiotics which may affect their stability in the environment, allowing some antibiotics to persist longer than others. Sensitivity against environmental factors, such as temperature, light, and moisture, differ by antibiotics, which may lead to the seasonal variation of antibiotic residues in surface water [54]. In addition, precipitation could impact the amounts of runoffs, such as animal manure, into the receiving waters, thus likely affecting the variability in antibiotic concentrations over time.

Antibiotic concentrations within a 10^0^ ng/L to 10^3^ ng/L range were commonly detected in surface water and WWTP effluents, with the concentrations more commonly in the low ng/L range and occasionally exceeding 10^3^ ng/L. This is consistent with the findings of the previous studies on surface water across the US and Canada [55,56,57]. Even though these antibiotic concentrations are several orders of magnitude lower than the concentrations used for therapeutic purposes, it has been shown that low antibiotic concentrations, much lower than their MICs, can select for resistance and enhance AR emergence [14]. In addition, the concentrations of the antibiotics in the water samples often exceeded the predicted no-effect concentrations (PNECs; the concentrations below which no adverse effects of exposure will most likely occur) for resistance selection [58]. This indicates that the antibiotics found in the aquatic environment have the potential to select for AR. Also, it should be noted that antibiotics are commonly diluted and degraded in the environment, and the actual antibiotic levels that organisms are exposed to in the environment may be greater than the levels detected, posing a greater selective pressure than is measured [59]. Therefore, the presence and widespread dissemination of antibiotics at their sub-MIC levels in the aquatic environment is a concern, and the release of antibiotics into the receiving water needs to be further evaluated.

### 3.2. WWTPs as a Source of AR Contaminants

Wastewater samples from three water reclamation facilities were included in this study to investigate if WWTPs are a source of AR in the natural environment. The results have shown that WWTPs only partially removed ARB, ARGs, and antibiotics, with the effluents containing high levels of AR contaminants as some previous studies have reported [60,61,62,63]. ARB, ARGs, and antibiotic residues present in the effluents would end up in rivers and streams and potentially affect the indigenous bacterial populations within the receiving waters. Wastewater treated by WWTPs has also been widely reused for different purposes, including agriculture and landscape irrigation and aquaculture. Hence, wastewater effluents should be treated further to prevent them from spreading into the environment.

Wastewater treatment greatly reduced the absolute copy numbers of ARGs but did not significantly change their relative copy numbers. Previous studies have also shown that while the absolute abundance of ARGs was efficiently reduced in WWTPs, their relative abundance was reduced to a lesser degree [62,64,65]. The absolute abundance of ARGs was potentially decreased due to a reduction in the overall abundance of bacterial populations during the treatment process, whereas a favorable environment for horizontal gene transfer of ARGs, enhanced by high density of bacteria and nutrients as well as antibiotics within the treatment system, could have led to a smaller reduction in the relative abundance of ARGs [66].

The removal efficiency of ARGs differed by season, with the highest efficiency in winter when gene abundance was highest. Similarly, others reported higher ARG reduction in winter compared with other seasons [67,68], while Jiao et al. observed higher removal efficiency of ARGs in summer than in winter [69]. Likewise, there are inconsistent reports on the seasonal pattern of antibiotic removal efficiency of WWTPs. A few studies showed that the total levels of antibiotics in wastewater influents were higher in winter, most probably due to increased use of antibiotics in winter, which could have lowered the removal efficiency of antibiotics in winter [70,71]. On the other hand, a contradictory finding was observed by Osinska et al.; although much higher antibiotic concentrations were detected in winter compared with fall, the reduction in the concentrations of antibiotics during the wastewater treatment was still higher in winter [68]. In contrast, the current study and a study by Shen et al. indicated that the total antibiotic concentration in wastewater influents was higher in summer than the other seasons while the removal efficiency of antibiotics was lowest in summer [72]. A study by Zielinski et al. detected the highest antibiotic concentrations in fall and also the lowest reduction of antibiotic concentrations in fall [73]. In most cases, the seasonal patterns observed in the antibiotic concentrations in wastewater tend to be associated with increased antibiotic administration as reflected by the seasonality of the infections among the populations.

Water samples collected upstream and downstream of the NORO WWTP were compared to evaluate the impact of treated effluent on surface waters. As the WWTPs included in this study are located near the end of the watershed within Athens city limits, more upstream water samples were collected than downstream water samples, making such a comparison possible only for the NORO WWTP. Previous studies have detected significantly higher rates and concentrations of ARB, ARGs, and antibiotics in water samples collected below the discharge point of treated effluents compared with upstream samples, implying that WWTPs are an important source of AR contamination of the aquatic environment [61,68,73,74,75,76]. However, while the WWTP effluent discharges contained high concentrations of ARGs and antibiotics, no evidence for differences in the ARGs and antibiotic concentrations was documented between the upstream water samples and downstream water samples in this study.

Many of the water sampling sites contaminated with AR contaminants were not associated with or located downstream of WWTPs. Only a small number of the sites received any treated wastewater input, but most of the sites contained high levels of AR contaminants throughout the year, indicating that there are other sources of AR pollution apart from WWTPs. Some studies also detected high concentrations of AR contaminants in upstream water samples [60,74]. While we only considered WWTPs as the source of AR contaminants in our study, non-point sources of pollution, such as recreational activities, run-off from agricultural land, sewer leaks, and septic tanks, could also be sources of ARB, ARGs, and antibiotics. Unlike human wastes that undergo waste treatment process, untreated animal wastes are commonly applied to agricultural land for soil fertilization, and as the runoff water reaches nearby surface water, it may pick up ARB originated from animal wastes [77]. In addition, untreated human waste may be released into the receiving water due to aged or poorly maintained septic systems or leaks from failing infrastructure. Approximately 70% of septic tanks in the city of Athens where this study was conducted (encompassing mainly Athens-Clarke County) are older than 25 years and 28% are older than 45 years, posing a potential environmental risk [78]. Aging or poorly maintained septic systems may fail to function properly and release raw sewage into the environment, increasing the potential for AR contamination in the receiving water. Indeed, a study conducted in the same watershed by Damashek et al. found that aging sewage and septic systems, rather than WWTPs, are primary sources of ARGs and fecal bacteria in this watershed [46]. It seems likely that these systems could also be sources for the antibiotics detected in the watershed. In addition, a sampling site within a lake that offers recreational activities (NORO 401) measured high levels of antibiotic residues in spring. This suggests that human and pet populations could contribute to AR contamination in surface water through recreational activities. Therefore, the effects of different sources of pollutions, other than treated wastewater from WWTPs, released into surface water need to be studied as well.

## 4. Materials and Methods

### 4.1. Surface Water and Wastewater Samples

**Figure 4:**
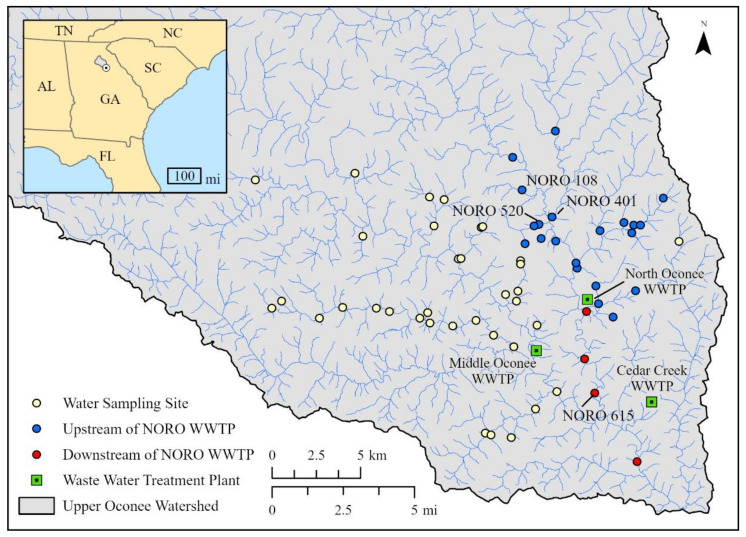
Map of water sampling sites in the Upper Oconee Watershed, Georgia, USA. Wastewater treatment plants (WWTPs) are shown as squares while water sampling sites are shown as circles, with sampling sites upstream of the North Oconee (NORO) WWTP symbolized as blue circles and sampling sites downstream of the NORO WWTP symbolized as red circles.

Water samples were collected from the rivers and streams located within the Upper Oconee Watershed in northeastern Georgia, USA (USGS Cataloging unit: 03070101). Sampling sites were located along the Middle Oconee River (MIDO), North Oconee River (NORO), Big Creek Oconee (BICO), and their tributaries (Figure 4). Water samples were collected seasonally four times between 2017 and 2018 (2017 fall through 2018 summer). The number of water samples varied from 41 to 44 during each collection date. Water samples were also collected from untreated influent and treated effluent from three WWTPs located within the watershed, i.e., Cedar Creek (Cedar WWTP), Middle Oconee (MIDO WWTP), and North Oconee (NORO WWTP) water reclamation facilities (Figure 4) on the same days. All water samples were stored at 4 °C until processed within 24 h of collection.

### 4.2. Antibiotic-Resistant E. coli, Enterococcus, and Salmonella

#### 4.2.1. Isolation and Identification of *E. coli*, *Enterococcus*, and *Salmonella*

Water samples (1 L) were filtered using cellulose filter powder (Aqua Dew^TM^, Lahore, Pakistan) and enriched in buffered peptone water (BPW; BD Difco^TM^, Franklin Lakes, NJ, USA) and incubated overnight at 37 °C as previously described [79]. All samples in this study were incubated at 37 °C for 18–24 h except when otherwise noted. Wastewater effluent samples were filtered and enriched in BPW using the same methods as water samples, while influent samples were not filtered or enriched.

All media used for the isolation and identification of bacteria were purchased from BD unless otherwise indicated. For *E. coli* isolation, 0.1 mL of each BPW enrichment was streaked on a CHROMagar ECC agar plate (CHROMagar, Paris, France). After the overnight incubation, one presumptive *E. coli* isolate per sample was confirmed using PCR as previously described [80]. Enterococci were isolated as previously described by streaking 0.1 mL of each BPW enrichment on an Enterococcosel agar plate for overnight incubation [25]. One presumptive *Enterococcus* isolate per sample was confirmed and the species was determined using multiplex PCR [81]. For *Salmonella* isolation, 1 mL of each BPW enrichment was transferred to Gram-negative Hajna (GN) and tetrathionate (Tet) broths for selective enrichment as previously described [23]. The secondary selective enrichment using Rappaport–Vassiliadis (RV) broth was followed by selective isolation using brilliant green sulfa (BGS) and xylose lysine tergitol 4 (XLT4) agar plates. One presumptive *Salmonella* colony was picked from each positive plate (up to four isolates per sample) and confirmed using triple sugar iron (TSI) and lysine iron agar (LIA) slants and also using *Salmonella* multiplex assay for rapid typing (SMART) PCR [82]. Samples collected from untreated influent were directly transferred to selective media for bacterial isolation and processed as described above.

#### 4.2.2. Antimicrobial Susceptibility Testing

MICs of all the confirmed bacterial isolates were determined by broth microdilution using the Sensititre™ (TREK Diagnostic Systems, Cleveland, OH, USA) semi-automated antimicrobial susceptibility system (TREK Diagnostic Systems) and the Sensititre™ National Antimicrobial Resistance Monitoring System (NARMS) plates (CMV3AGNF for *E. coli* and *Salmonella* and CMV3AGPF for *Enterococcus*) according to manufacturer’s directions. *E. coli* (n = 191) and *Salmonella* (n = 328) isolates were tested against 14 antibiotic agents and *Enterococcus* (n = 191) isolates were tested against 16 antibiotic agents (Table S10). Each isolate was classified as resistant, intermediate, or susceptible using the breakpoints set by Clinical and Laboratory Standards Institute (CLSI) [83]. Where no CLSI breakpoints were established, those defined by NARMS were used (https://www.ars.usda.gov/ARSUserFiles/60400520/NARMS/ABXSalm.pdf (accessed on 14 December 2020); https://www.ars.usda.gov/ARSUserFiles/60400520/NARMS/ABXEntero.pdf (accessed on 14 December 2020)). For azithromycin, without CLSI-approved breakpoints, a MIC of ≥32 μg/mL was used [84]. *E. coli* ATCC 25922, *P. aeruginosa* ATCC 27853, *Enterococcus faecalis* ATCC 29212, and *S. aureus* ATCC 29213 were used as control strains for MIC determination. For the analysis, isolates identified as intermediate were considered susceptible to the drug.

### 4.3. ESBL-Producing Enterobacteriaceae and CRE

#### 4.3.1. Isolation and Identification of ESBL-Producing *Enterobacteriaceae* and CRE

For the isolation of ESBL producers, 100 mL of water samples were vacuum filtered in duplicate onto mixed cellulose ester membranes of a 47 mm diameter with a 0.45 μm pore size (Millipore Sigma, Burlington, MA, USA). The membranes were then placed onto HiCrome ESBL agar plates containing ESBL selective supplements (HiMedia, Mumbai, India) for the 2017 fall collection and onto CHROMagar ESBL agar plates (CHROMagar) for the subsequent collections. Additionally, for the subsequent collections, 100 mL of each water sample was filtered, and membranes were placed in 9 mL of BPW. After the overnight incubation, a loopful of overnight culture was streaked onto CHROMagar ESBL and CHROMagar mSuperCARBA agar plates (CHROMagar) and incubated overnight for the detection of ESBL producers and CRE, respectively. Up to four well-isolated blue (presumptive *Klebsiella*, *Enterobacter*, or *Citrobacter*) and pink (presumptive *E. coli*) colonies per plate were selected from directly plated ESBL media as well as from ESBL and CRE media plated with enriched cultures. Presumptive ESBL producers and CRE were identified to species using the VITEK^®^ 2 system and VITEK^®^ 2 GN ID cards (bioMérieux, Durham, NC, USA) according to the manufacturer’s directions.

#### 4.3.2. Confirmation of ESBL Producers and CRE and Detection of β-Lactamase Genes

All presumptive ESBL-producing *Enterobacteriaceae* and CRE were tested by PCR to detect 11 β-lactamase genes according to the given references: *bla*_CMY-2_ [85], *bla*_CTX-M_ [86], *bla*_KPC_ [87,88], *bla*_NDM-1_ [87,88], *bla*_OXA-1_ [89], *bla*_SHV_ [90], *bla*_TEM_ [91], *bla*_IMI-NmcA_ [92], *bla*_IMP_ [93], *bla*_VEB_ [94], and *bla*_VIM_ [87,88]. Isolates carrying at least one β-lactamase gene were screened for ESBL production and their MICs against 16 antibiotics (Table S10) were determined by broth microdilution as described above using Sensititre™ ESBL plates (ESB1F) (TREK Diagnostic Systems). The control strains included *K. pneumoniae* ATCC 700,603 in addition to the four aforementioned controls. *Enterobacteriaceae* isolates were considered ESBL producers if they were resistant to cefotaxime and/or ceftazidime and exhibited a ≥3-two-fold decrease in MIC of cefotaxime/clavulanic acid compared with cefotaxime alone, and/or a ≥3-two-fold decrease in MIC of ceftazidime/clavulanic acid compared with ceftazidime alone [83]. *Enterobacteriaceae* isolates were considered CRE if resistant to imipenem and/or meropenem (both with breakpoints > 4 μg/mL).

### 4.4. Antibiotic Resistance Gene Markers

Real-time PCR (qPCR) analyses were conducted to quantify bacterial 16S rRNA genes (for relative gene abundances) and six ARGs, namely *erm*B (macrolide-lincosamide-streptogramin B), *tet*B (tetracycline), *qnr*S (quinolone), *bla*_CTX-M_ (β-lactam), *bla*_SHV_ (β-lactam), and *bla*_KPC_ (β-lactam), using the TaqMan^TM^ Environmental Master Mix 2.0 (Thermo Fisher Scientific, Waltham, MA, USA) as described by Damashek et al. [46]. Synthetic gBlock gene fragment standards were run for each assay, and an internal TaqMan^TM^ Exogenous Internal Positive Control was used to assess inter-run variability. Gene abundances were reported as gene copies/mL of water. Detailed methods and qPCR data were previously reported by Damashek et al. [46].

### 4.5. Antibiotics

#### 4.5.1. Antibiotics

A total of 26 antibiotics, representing 14 classes were selected for this study: aminoglycoside [gentamicin, kanamycin, streptomycin], carbapenem [meropenem], cephalosporin [ceftazidime, ceftiofur, ceftriaxone], dihydrofolate reductase (DHFR) inhibitors [trimethoprim], glycopeptide [vancomycin], glycylcycline [tigecycline], lincosamide [lincomycin], lipopeptide [daptomycin], macrolide [azithromycin, erythromycin, tylosin], oxazolidinone [linezolid], penicillins [amoxicillin, ampicillin, methicillin, oxacillin, penicillin], quinolone [nalidixic acid, ciprofloxacin], sulfonamide [sulfamethoxazole, sulfisoxazole], and tetracycline [tetracycline].

#### 4.5.2. Sample Preparation

Each water sample was filtered through a 0.2 µm filter to remove any biologicals and stored at 4 °C. Within 24–48 h after filtering, each sample was spiked with 5 μL of internal standard (deuterated caffeine, imidacloprid, and diphenhydramine) before extraction on a preconditioned (6 mL methanol then 6 mL of water) Oasis^®^ hydrophilic–lipophilic balance (HLB; 6 cc, 500 mg, Waters, Milford, MA, USA) solid-phase extraction (SPE) cartridges. Afterwards, the cartridges were dried and stored in a −20 °C freezer until elution. Antibiotics were eluted sequentially with 6 mL methanol and 6 mL dichloromethane, then gently evaporated under nitrogen gas, and reconstituted in 10% acetonitrile for LC-MS/MS analysis.

#### 4.5.3. LC-MS/MS Analysis

A TSQ Quantum Ultra™ accurate mass triple quadrupole mass spectrometer (Thermo Fisher Scientific) with an electrospray interface was used for the detection of antibiotics. Separation of compounds was achieved using a Kinetex^®^ 2.6 μM C18 (100 Å, 150 × 2.1 mm) high-performance liquid chromatography (HPLC) column (Phenomenex, Torrance, CA, USA). Injection volume for all samples was 20 μL. All samples were run with blanks and calibration curves at the beginning and end of the sequence with quality assurance/quality control (QA/QC) analyzed every 10 samples. The mobile phase A consisted of 0.1% formic acid in water and mobile phase B consisted of 0.1% formic acid in acetonitrile. Initial conditions were 0% B for 4 min, ramped to 60% B over 16 min, increased to 98% B, and held for 4 min before returning to starting conditions (total run time = 30 min) with a flow rate of 200 μL/min. Quantification of all 26 antibiotics was performed in positive mode with multiple reaction monitoring (MRM). Instrument parameters were optimized for each compound prior to analysis (Table S11).

### 4.6. Statistical Analysis Methods

Bayesian generalized linear mixed models (GLMM) were fitted to the data from both surface water and wastewater samples. For presence–absence data of AR contaminants in surface water, a logistic GLMM was fitted with season, contaminant, and their interactions as fixed predictors. A separate logistic GLMM was fit for β-lactamase-containing bacteria pooled together. For copy numbers of ARGs in surface water, a GLMM was fitted with a hurdle gamma response distribution. For wastewater samples, we did not fit a formal statistical model to the presence–absence data due to the sparsity of presences for individual AR contaminants. Another hurdle gamma GLMM was fitted with sample type (influent and effluent), gene, season, and their interactions as fixed predictors to assess the ability of WWTPs to reduce ARG concentrations in wastewater. A similar model was fit for antibiotic concentration with sample type, drug, season, and their interactions as predictors. Additional models were fitted with the interaction of WWTP and sample type as predictors to compare the effectiveness of the treatment plants. Finally, models were fit comparing ARG copy numbers and antibiotic concentrations upstream and downstream of the NORO WWTP.

In all cases, marginal means and trends were estimated using the medians of the posterior predictive distributions and uncertainty was characterized with the 66%, 90%, 95%, and 99% quantile credible intervals (QCI). These marginal means were contrasted by taking the ratio between different groups, again finding the median and QCI of the posterior distributions of the ratios. A complete description of the statistical methods, including model fitting details and information about software used, is found in the supplementary file (File S1).

## 5. Conclusions

This study endeavored to provide an overall picture of AR, including ARB, ARGs, and antibiotics, in a mixed-use watershed. The current study has shown that antibiotic-related contaminants are prevalent in the freshwater environment, including commensal and pathogenic bacteria that are resistant to antibiotics used for human and veterinary purposes, medically important antibiotics, as well as the genes associated with resistance to these antibiotics. The wide dissemination and abundance of such AR contaminants may potentially pose health concerns to the populations exposed to contaminants in these water sources. ESBL-, AmpC-, and carbapenemase-producing *Enterobacteriaceae*, which are increasingly reported in healthcare and community settings, were commonly found in surface water and wastewater effluents that were released into this water, indicating their prevalence in the community as well as the natural environment. These findings suggest that continuous surveillance of AR contaminants in the aquatic environment, identification of AR hotspots, and preventive interventions are needed to ensure public health. The comparison of wastewater influent and effluent samples has shown that wastewater treatment is not effective in removing all the AR contaminants present in the wastewater and, in fact, some antibiotics and resistance genes were enriched during the process. Treated effluents carried these AR contaminants, indicating that WWTPs are a source of AR in the receiving water. However, surface water samples that had not been impacted by WWTPs also had high levels of AR contaminants, suggesting that WWTPs are not the sole contributors of AR in the environment, and other sources of AR contaminants should also be considered. In our subsequent study, we will investigate the factors that could have led to high prevalence and widespread distribution of AR contaminants in the water.

## Figures and Tables

**Table 1:** Recovery rates of antibiotic-resistant *Salmonella*, *E. coli*, and *Enterococcus* from surface water and wastewater.

Source	Season (No. of Samples)	*Salmonella*		*E. coli*		*Enterococcus*	
		% of Positive Samples (No. of Isolates)	No. of AR Isolates (No. of MDR Isolates) ^1^	% of Positive Samples (No. of Isolates)	No. of AR Isolates (No. of MDR Isolates) ^1^	% of Positive Samples (No. of Isolates)	No. of AR Isolates (No. of MDR Isolates) ^1^
Surface water	2017 fall (43)	72.1 (78)	27 (27)	100 (43)	3 (0)	100 (43)	43 (2)
	2018 winter (41)	48.8 (57)	0 (0)	100 (41)	4 (2)	97.6 (40)	40 (4)
	2018 spring (42)	59.5 (74)	2 (0)	100 (42)	2 (1)	100 (42)	42 (1)
	2018 summer (44)	93.2 (94)	0 (0)	100 (44)	2 (1)	100 (44)	44 (1)
Influent	2017 fall (3)	100.0 (5)	2 (0)	100.0 (3)	2 (2)	100 (3)	3 (0)
	2018 winter (3)	100.0 (5)	2 (0)	100.0 (3)	0 (0)	100 (3)	3 (1)
	2018 spring (3)	100.0 (7)	2 (0)	100 (3)	0 (0)	100 (3)	3 (1)
	2018 summer (3)	100.0 (6)	0 (0)	100 (3)	0 (0)	100 (3)	3 (0)
Effluent	2017 fall (3)	0 (0)	0 (0)	66.7 (2)	0 (0)	100 (3)	3 (1)
	2018 winter (2)	0 (0)	0 (0)	100.0 (2)	1 (1)	100 (2)	2 (0)
	2018 spring (3)	0 (0)	0 (0)	100 (3)	1 (0)	100 (3)	3 (1)
	2018 summer (3)	33.3 (2)	0 (0)	66.7 (2)	1 (1)	66.7 (2)	2 (1)

^1^ Abbreviations: AR, antibiotic resistant; MDR, multidrug resistant.

**Table 2:** β-lactamase-positive *Enterobacteriaceae*, ESBL-producing *Enterobacteriaceae*, and CRE from surface water and wastewater.

Season	Source (No. of Samples)	Organisms	No. of β-Lactamase-Positive *Enterobacteriaceae*	No. of ESBL-Producing *Enterobacteriaceae* ^1^	No. of CRE ^1^
2017 fall	water (42)	*Escherichia coli*	3	2	0
	influent (3)	*Enterobacter* spp.	2	0	1
	effluent (3)	*Escherichia coli*	1	1	0
2018 winter	water (42)	*Escherichia coli*	1	1	0
		*Serratia fonticola*	19	19	2
	influent (3)	*Citrobacter freundii*	1	0	0
		*Escherichia coli*	2	1	0
	effluent (3)	*Enterobacter asburiae*	1	0	1
2018 spring	water (41)	*Escherichia coli*	3	2	0
		*Klebsiella oxytoca*	1	0	0
		*Klebsiella pneumoniae*	3	2	1
		*Serratia fonticola*	40	1	2
	influent (3)	*Escherichia coli*	1	1	0
		*Kluyvera ascorbata*	1	0	0
		*Kluyvera cryocrescens*	2	2	0
	effluent (3)	*Citrobacter braakii/freundii*	1	0	0
		*Klebsiella pneumoniae*	3	2	0
2018 summer	water (43)	*Serratia fonticola*	38	0	2
		*Enterobacter cloacae* complex	7	0	7
		*Escherichia coli*	4	3	0
		*Klebsiella pneumoniae*	1	1	0
	influent (3)	*Enterobacter asburiae*	1	0	0
		*Enterobacter cloacae* complex	1	0	0
		*Escherichia coli*	3	3	0
		*Klebsiella pneumoniae*	3	1	0
		*Kluyvera cryocrescens*	1	0	0
	effluent (3)	*Enterobacter cloacae* complex	2	0	2
		*Klebsiella pneumoniae*	1	0	0

^1^ Abbreviations: ESBL, extended-spectrum β-lactamase; CRE, carbapenem-resistant *Enterobacteriaceae*.

**Table 3:** Number of samples positive for ARGs using qPCR and ARG copy numbers in surface water, influent, and effluent samples.

**Season**	**Source (No. of Samples)**	***erm*B**			***tet*B**			***bla*_KPC_**		
		**No. of Positive Samples (%)**	**Maximum (Copies/mL)**	**Average (Copies/mL)**	**No. of Positive Samples (%)**	**Maximum (Copies/mL)**	**Average (Copies/mL)**	**No. of Positive Samples (%)**	**Maximum (Copies/mL)**	**Average (Copies/mL)**
2017 fall	surface water (38)	23 (60.5)	1533.8	57.2	10 (26.3)	127.0	4.0	9 (23.7)	13.5	1.6
	influent (3)	3 (100)	189,008.9	125,110.9	3 (100)	4591.6	2524.8	3 (100)	299,148.9	153,214.4
	effluent (3)	3 (100)	33,052.9	11,394.3	3 (100)	909.1	311.7	3 (100)	31,100.5	11,630.9
2018 winter	surface water (38)	8 (21.6)	355.0	11.3	2 (5.6)	0.7	0.0	5 (13.5)	49.9	2.0
	influent (3)	3 (100)	338,120.7	213,492.4	3 (100)	15,694.5	8238.5	3 (100)	362,358.8	142,942.9
	effluent (3)	3 (100)	277.9	182.7	2 (66.7)	5.3	2.1	3 (100)	197.5	115.7
2018 spring	surface water (34)	8 (23.5)	41.0	1.9	1 (2.9)	1.6	0.0	2 (5.9)	76.2	2.4
	influent (3)	3 (100)	98,933.2	46,165.2	3 (100)	3219.8	1216.4	3 (100)	73,007.5	48,650.3
2018 summer	surface water (40)	11 (27.5)	347.5	11.9	2 (5.0)	1.0	0.1	7 (17.5)	377.5	9.8
**Season**	**Source (No. of Samples)**	***bla*_SHV_**			***qnr*S**			***bla*_CTX-M_**		
		**No. of positive samples (%)**	**Maximum (copies/mL)**	**Average (copies/mL)**	**No. of positive samples (%)**	**Maximum (copies/mL)**	**Average (copies/mL)**	**No. of positive samples (%)**	**Maximum (copies/mL)**	**Average (copies/mL)**
2017 fall	surface water (38)	9 (23.7)	325.0	10.7	8 (21.1)	308.4	12.5	3 (7.9)	455.2	15.9
	influent (3)	3 (100)	37,087.9	24,498.7	3 (100)	624,275.8	418,281.0	3 (100)	6,740.0	2633.0
	effluent (3)	3 (100)	7423.3	2637.1	3 (100)	138,990.3	50,771.0	2 (66.7)	1499.5	502.4
2018 winter	surface water (38)	2 (5.4)	2.0	0.1	8 (21.1)	582.6	19.5	0 (0.0)	0.0	0.0
	influent (3)	3 (100)	40,126.5	26,100.6	3 (100)	605,863.5	322,060.7	3 (100)	26,050.7	13,449.5
	effluent (3)	3 (100)	37.1	16.6	3 (100)	1784.4	942.9	1 (33.3)	51.8	17.3
2018 spring	surface water (34)	1 (2.9)	2.0	0.1	3 (9.4)	122.4	4.6	0 (0.0)	0.0	0.0
	influent (3)	3 (100)	34,286.7	13,942.2	3 (100)	291,963.4	137,383.9	3 (100)	26,165.9	9461.5
2018 summer	surface water (40)	2 (5.0)	7.7	0.2	8 (20.0)	703.0	21.5	0 (0.0)	0.0	0.0

**Table 4:** Antibiotic concentrations in surface water and wastewater by season.

**Season**	**Source (No. of Samples)**		**Antibiotics ^1^**												
			**AMX**	**AMP**	**AZI**	**AXO**	**TAZ**	**TIO**	**CIP**	**DAP**	**ERY**	**GEN**	**KAN**	**LIN**	**LNZ**
2017 fall	water (43)	maximum concentrations (ng/L)	45.2	44.6	106.6	0.0	10.9	34.9	13.5	0.0	26.1	0.0	0.0	39.9	37.7
		average concentrations (ng/L)	2.1	4.4	3.9	0.0	0.3	1.4	1.6	0.0	1.1	0.0	0.0	1.3	2.4
		no. of times detected	14	15	7	0	1	21	28	0	19	0	0	14	9
	wastewater (6)	maximum concentrations (ng/L)	2.1	52.8	120.6	0.0	0.0	38.0	123.7	16.3	20.8	0.0	0.0	1.3	0.6
		average concentrations (ng/L)	0.4	13.9	29.2	0.0	0.0	6.7	49.8	2.7	11.2	0.0	0.0	0.6	0.2
		no. of times detected	1	5	6	0	0	3	6	1	5	0	0	6	3
2018 winter	water (30)	maximum concentrations (ng/L)	14.6	41.0	0.4	6.6	152.3	19.9	1.8	1472.8	0.1	122.0	0.1	3.8	0.1
		average concentrations (ng/L)	1.8	4.5	0.0	1.0	11.4	3.4	0.1	51.1	0.0	16.5	0.0	0.1	0.0
		no. of times detected	8	6	2	6	4	11	8	5	4	13	1	3	3
	wastewater (6)	maximum concentrations (ng/L)	91.0	18.3	22.9	21.9	686.0	37.8	4.6	1292.6	86.3	199.6	0.0	4.6	1.5
		average concentrations (ng/L)	15.2	3.1	6.8	3.6	162.0	15.5	0.8	237.1	23.0	48.1	0.0	1.4	0.3
		no. of times detected	1	1	3	1	2	3	1	2	3	2	0	3	2
2018 spring	water (39)	maximum concentrations (ng/L)	67.6	293.3	31.9	750.5	524.3	19.8	14.9	167.8	21.9	37.9	12.0	259.7	13.7
		average concentrations (ng/L)	3.8	15.4	0.9	49.1	17.3	3.4	3.1	10.8	1.4	1.0	0.3	19.4	0.9
		no. of times detected	12	14	19	6	3	17	19	7	14	1	1	12	18
	wastewater (6)	maximum concentrations (ng/L)	6.9	216.9	46.7	0.0	0.0	14.7	67.1	0.0	113.2	0.0	0.0	59.0	4.5
		average concentrations (ng/L)	1.5	74.9	18.8	0.0	0.0	2.5	30.4	0.0	36.6	0.0	0.0	9.8	2.0
		no. of times detected	2	4	5	0	0	1	4	0	3	0	0	1	4
2018 summer	water (39)	maximum concentrations (ng/L)	39.9	4.6	26.9	8.6	23.8	12.7	13.6	2.9	66.5	23.7	24.9	0.1	27.3
		average concentrations (ng/L)	12.6	0.6	6.3	0.5	1.8	1.0	1.9	0.3	13.2	3.0	1.9	0.0	5.8
		no. of times detected	31	8	10	6	3	9	11	7	20	5	3	1	10
	wastewater (6)	maximum concentrations (ng/L)	43.6	4.3	326.5	0.0	31.0	25.7	29.7	19.1	3550.6	23.8	75.7	0.0	69.2
		average concentrations (ng/L)	10.9	0.7	121.6	0.0	9.4	6.5	8.9	3.2	978.7	4.0	23.1	0.0	40.0
		no. of times detected	2	1	3	0	2	2	2	1	3	1	3	0	6
**Season**	**Source (No. of Samples)**		**Antibiotics ^1^**												
			**MRP**	**MET**	**NAL**	**OXA**	**PEN**	**SMX**	**FIS**	**STR**	**TGC**	**TMP**	**TET**	**TYL**	**VAN**
2017 fall	water (43)	maximum concentrations (ng/L)	19.6	31.5	13.6	80.9	40.9	66.7	25.4	44.9	0.0	40.9	0.0	22.1	0.0
		average concentrations (ng/L)	0.9	2.1	0.6	10.1	2.4	3.2	1.3	6.4	0.0	2.9	0.0	1.4	0.0
		no. of times detected	10	14	9	12	13	25	16	16	0	42	0	18	0
	wastewater (6)	maximum concentrations (ng/L)	0.6	6.2	14.3	163.3	26.8	173.5	43.5	0.0	0.0	70.7	0.0	2.0	0.0
		average concentrations (ng/L)	0.1	1.6	4.1	37.0	5.4	72.6	10.0	0.0	0.0	29.2	0.0	0.6	0.0
		no. of times detected	1	2	3	2	3	6	4	0	0	6	0	3	0
2018 winter	water (30)	maximum concentrations (ng/L)	3.9	0.6	0.6	502.6	0.4	4.0	0.1	26.5	0.0	0.7	0.3	0.8	0.2
		average concentrations (ng/L)	0.3	0.0	0.0	17.0	0.0	0.2	0.0	3.5	0.0	0.2	0.0	0.2	0.0
		no. of times detected	6	6	2	2	1	7	10	5	0	20	4	20	1
	wastewater (6)	maximum concentrations (ng/L)	0.0	2.1	0.2	10.6	0.0	35.0	0.0	28.2	0.0	91.5	2.8	91.6	0.0
		average concentrations (ng/L)	0.0	0.4	0.0	1.8	0.0	18.5	0.0	9.2	0.0	16.1	1.1	26.1	0.0
		no. of times detected	0	1	1	1	0	6	0	2	0	4	4	6	0
2018 spring	water (39)	maximum concentrations (ng/L)	220.7	50.0	13.7	593.2	19.7	29.0	60.5	159.3	46.5	16.6	44.8	63.6	238.0
		average concentrations (ng/L)	8.1	4.3	0.4	23.6	3.9	1.2	4.7	6.9	3.5	4.0	15.8	6.0	18.4
		no. of times detected	13	17	2	5	17	9	12	9	5	36	23	20	8
	wastewater (6)	maximum concentrations (ng/L)	11.8	42.0	148.0	0.0	99.7	362.3	85.8	256.7	288.4	168.9	490.0	12.8	262.8
		average concentrations (ng/L)	2.7	8.2	25.8	0.0	28.3	170.1	23.0	62.9	84.9	100.1	173.5	3.3	112.1
		no. of times detected	2	2	4	0	4	6	4	2	3	6	5	3	3
2018 summer	water (39)	maximum concentrations (ng/L)	9.9	21.6	81.8	0.0	23.8	98.4	2.8	0.0	0.0	87.5	0.2	24.5	0.0
		average concentrations (ng/L)	0.7	2.2	4.5	0.0	3.7	11.6	0.5	0.0	0.0	19.3	0.0	9.8	0.0
		no. of times detected	4	12	4	0	12	32	11	0	0	37	1	33	0
	wastewater (6)	maximum concentrations (ng/L)	31.8	28.2	328.5	0.0	0.0	2193.5	19.0	0.0	0.0	5777.0	20.9	134.0	0.0
		average concentrations (ng/L)	5.3	4.7	113.8	0.0	0.0	909.6	3.2	0.0	0.0	1228.0	3.7	55.2	0.0
		no. of times detected	1	1	4	0	0	6	1	0	0	6	2	6	0

^1^ Antibiotics: amoxicillin (AMX), ampicillin (AMP), azithromycin (AZI), ceftriaxone (AXO), ceftazidime (TAZ), ceftiofur (TIO), ciprofloxacin (CIP), daptomycin (DAP), erythromycin (ERY), gentamicin (GEN), kanamycin (KAN), lincomycin (LIN), linezolid (LNZ), meropenem (MRP), methicillin (MET), nalidixic acid (NAL), oxacillin (OXA), penicillin (PEN), sulfamethoxazole (SMX), sulfisoxazole (FIS), streptomycin (STR), tigecycline (TGC), trimethoprim (TMP), tetracycline (TET), tylosin (TYL), and vancomycin (VAN).

## Data Availability

The data presented in this study are available on request from the corresponding author.

## Supplementary Materials

The following supporting information can be downloaded at: https://www.mdpi.com/article/10.3390/antibiotics12111586/s1, Table S1: Masterfile for 2017 fall sampling; Table S2: Masterfile for 2018 winter sampling; Table S3: Masterfile for 2018 spring sampling; Table S4: Masterfile for 2018 summer sampling; Table S5: β-lactamase gene patterns in ESBL and CRE isolates recovered from surface water and wastewater; Table S6: Seasonal contrasts for the presence of (a) ESBL- and (b) β-lactamase-producing bacteria in surface water; Table S7: Estimated (a) absolute and (b) relative ARG copy numbers in wastewater influent and effluent; Table S8: Estimated antibiotic concentrations in wastewater influent and effluent; Table S9: Estimated (a) ARG copy numbers and (b) antibiotic concentration in upstream and downstream water samples; Table S10: Antibiotics used for MIC determination of bacterial isolates and their breakpoints; Table S11: Antibiotics used for detection in water samples and the instrument parameters optimized for each compound prior to analysis; File S1: A complete description of the statistical methods. References [95,96,97,98,99,100,101] are cited in the supplementary materials.

## References

[1] Wellington E.M., Boxall A.B., Cross P., Feil E.J., Gaze W.H., Hawkey P.M., Johnson-Rollings A.S., Jones D.L., Lee N.M., Otten W. (2013). The role of the natural environment in the emergence of antibiotic resistance in gram-negative bacteria. Lancet Infect. Dis..

[2] Kusi J., Ojewole C.O., Ojewole A.E., Nwi-Mozu I. (2022). Antimicrobial Resistance Development Pathways in Surface Waters and Public Health Implications. Antibiotics.

[3] Singh A.K., Kaur R., Verma S., Singh S. (2022). Antimicrobials and Antibiotic Resistance Genes in Water Bodies: Pollution, Risk, and Control. Front. Environ. Sci..

[4] Cho S., Jackson C.R., Frye J.G. (2023). Freshwater environment as a reservoir of extended-spectrum β-lactamase-producing Enterobacteriaceae. J. Appl. Microbiol..

[5] Cabello F.C. (2006). Heavy use of prophylactic antibiotics in aquaculture: A growing problem for human and animal health and for the environment. Environ. Microbiol..

[6] Li W., Zhang G. (2022). Detection and various environmental factors of antibiotic resistance gene horizontal transfer. Environ. Res..

[7] Thomson K.S. (2010). Extended-spectrum-beta-lactamase, AmpC, and Carbapenemase issues. J. Clin. Microbiol..

[8] Pitout J.D.D., Laupland K.B. (2008). Extended-spectrum β-lactamase-producing Enterobacteriaceae: An emerging public-health concern. Lancet Infect. Dis..

[9] Paterson D.L., Bonomo R.A. (2005). Extended-spectrum beta-lactamases: A clinical update. Clin. Microbiol. Rev..

[10] Hammoudi Halat D., Ayoub Moubareck C. (2020). The Current Burden of Carbapenemases: Review of Significant Properties and Dissemination among Gram-Negative Bacteria. Antibiotics.

[11] Sarmah A.K., Meyer M.T., Boxall A.B. (2006). A global perspective on the use, sales, exposure pathways, occurrence, fate and effects of veterinary antibiotics (VAs) in the environment. Chemosphere.

[12] Verlicchi P., Al Aukidy M., Zambello E. (2012). Occurrence of pharmaceutical compounds in urban wastewater: Removal, mass load and environmental risk after a secondary treatment—A review. Sci. Total Environ..

[13] Zhang Q.Q., Ying G.G., Pan C.G., Liu Y.S., Zhao J.L. (2015). Comprehensive evaluation of antibiotics emission and fate in the river basins of China: Source analysis, multimedia modeling, and linkage to bacterial resistance. Environ. Sci. Technol..

[14] Gullberg E., Cao S., Berg O.G., Ilback C., Sandegren L., Hughes D., Andersson D.I. (2011). Selection of resistant bacteria at very low antibiotic concentrations. PLoS Pathog..

[15] Liu A., Fong A., Becket E., Yuan J., Tamae C., Medrano L., Maiz M., Wahba C., Lee C., Lee K. (2011). Selective advantage of resistant strains at trace levels of antibiotics: A simple and ultrasensitive color test for detection of antibiotics and genotoxic agents. Antimicrob. Agents Chemother..

[16] Lundstrom S.V., Ostman M., Bengtsson-Palme J., Rutgersson C., Thoudal M., Sircar T., Blanck H., Eriksson K.M., Tysklind M., Flach C.F. (2016). Minimal selective concentrations of tetracycline in complex aquatic bacterial biofilms. Sci. Total Environ..

[17] Jutkina J., Marathe N.P., Flach C.F., Larsson D.G.J. (2018). Antibiotics and common antibacterial biocides stimulate horizontal transfer of resistance at low concentrations. Sci. Total Environ..

[18] Jutkina J., Rutgersson C., Flach C.F., Joakim Larsson D.G. (2016). An assay for determining minimal concentrations of antibiotics that drive horizontal transfer of resistance. Sci. Total Environ..

[19] Kohanski M.A., DePristo M.A., Collins J.J. (2010). Sublethal antibiotic treatment leads to multidrug resistance via radical-induced mutagenesis. Mol. Cell.

[20] Chow L., Waldron L., Gillings M.R. (2015). Potential impacts of aquatic pollutants: Sub-clinical antibiotic concentrations induce genome changes and promote antibiotic resistance. Front. Microbiol..

[21] Rizzo L., Manaia C., Merlin C., Schwartz T., Dagot C., Ploy M.C., Michael I., Fatta-Kassinos D. (2013). Urban wastewater treatment plants as hotspots for antibiotic resistant bacteria and genes spread into the environment: A review. Sci. Total Environ..

[22] Food and Drug Administration (FDA) (2008). National Antimicrobial Resistance Monitoring System—Enteric Bacteria (NARMS): 2004 Executive Report.

[23] Cho S., Hiott L.M., House S.L., Woodley T.A., McMillan E.A., Sharma P., Barrett J.B., Adams E.S., Brandenburg J.M., Hise K.B. (2022). Analysis of Salmonella enterica Isolated from a Mixed-Use Watershed in Georgia, USA: Antimicrobial Resistance, Serotype Diversity, and Genetic Relatedness to Human Isolates. Appl. Environ. Microbiol..

[24] Cho S., Hiott L.M., Woodley T.A., Frye J.G., Jackson C.R. (2020). Evaluation of a new chromogenic agar for the detection of environmental Enterococcus. J. Microbiol. Methods.

[25] Cho S., Hiott L.M., McDonald J.M., Barrett J.B., McMillan E.A., House S.L., Adams E.S., Frye J.G., Jackson C.R. (2020). Diversity and antimicrobial resistance of Enterococcus from the Upper Oconee Watershed, Georgia. J. Appl. Microbiol..

[26] Cho S., Nguyen H.A.T., McDonald J.M., Woodley T.A., Hiott L.M., Barrett J.B., Jackson C.R., Frye J.G. (2019). Genetic Characterization of Antimicrobial-Resistant Escherichia coli Isolated from a Mixed-Use Watershed in Northeast Georgia, USA. Int. J. Environ. Res. Public. Health.

[27] Raphael E., Wong L.K., Riley L.W. (2011). Extended-spectrum Beta-lactamase gene sequences in gram-negative saprophytes on retail organic and nonorganic spinach. Appl. Environ. Microbiol..

[28] Tanner W.D., VanDerslice J.A., Goel R.K., Leecaster M.K., Fisher M.A., Olstadt J., Gurley C.M., Morris A.G., Seely K.A., Chapman L. (2019). Multi-state study of Enterobacteriaceae harboring extended-spectrum beta-lactamase and carbapenemase genes in U.S. drinking water. Sci. Rep..

[29] Blaak H., van Hoek A.H., Veenman C., Docters van Leeuwen A.E., Lynch G., van Overbeek W.M., de Roda Husman A.M. (2014). Extended spectrum ss-lactamase- and constitutively AmpC-producing Enterobacteriaceae on fresh produce and in the agricultural environment. Int. J. Food Microbiol..

[30] Van Hoek A.H., Veenman C., van Overbeek W.M., Lynch G., de Roda Husman A.M., Blaak H. (2015). Prevalence and characterization of ESBL- and AmpC-producing Enterobacteriaceae on retail vegetables. Int. J. Food Microbiol..

[31] Kenzaka T., Tani K. (2017). Draft Genome Sequence of Extended-Spectrum Beta-Lactamase-Producing Serratia fonticola BWK15 Isolated from Feces of Anas penelope. Genome Announc..

[32] Richter L., Du Plessis E.M., Duvenage S., Korsten L. (2019). Occurrence, Identification, and Antimicrobial Resistance Profiles of Extended-Spectrum and AmpC beta-Lactamase-Producing Enterobacteriaceae from Fresh Vegetables Retailed in Gauteng Province, South Africa. Foodborne Pathog. Dis..

[33] Henriques I., Juca Ramos R.T., Barauna R.A., de Sa P.H., Marinho Almeida D., Carneiro A.R., Barbosa S., Pereira A., Alves A., Saavedra M.J. (2013). Draft Genome Sequence of Serratia fonticola UTAD54, a Carbapenem-Resistant Strain Isolated from Drinking Water. Genome Announc..

[34] Olson A.B., Silverman M., Boyd D.A., McGeer A., Willey B.M., Pong-Porter V., Daneman N., Mulvey M.R. (2005). Identification of a progenitor of the CTX-M-9 group of extended-spectrum beta-lactamases from Kluyvera georgiana isolated in Guyana. Antimicrob. Agents Chemother..

[35] Humeniuk C., Arlet G., Gautier V., Grimont P., Labia R., Philippon A. (2002). Beta-lactamases of Kluyvera ascorbata, probable progenitors of some plasmid-encoded CTX-M types. Antimicrob. Agents Chemother..

[36] Wilson B.M., El Chakhtoura N.G., Patel S., Saade E., Donskey C.J., Bonomo R.A., Perez F. (2017). Carbapenem-Resistant Enterobacter cloacae in Patients from the US Veterans Health Administration, 2006–2015. Emerg. Infect. Dis..

[37] Annavajhala M.K., Gomez-Simmonds A., Uhlemann A.C. (2019). Multidrug-Resistant Enterobacter cloacae Complex Emerging as a Global, Diversifying Threat. Front. Microbiol..

[38] Sugawara Y., Hagiya H., Akeda Y., Aye M.M., Myo Win H.P., Sakamoto N., Shanmugakani R.K., Takeuchi D., Nishi I., Ueda A. (2019). Dissemination of carbapenemase-producing Enterobacteriaceae harbouring blaNDM or blaIMI in local market foods of Yangon, Myanmar. Sci. Rep..

[39] Boyd D.A., Mataseje L.F., Davidson R., Delport J.A., Fuller J., Hoang L., Lefebvre B., Levett P.N., Roscoe D.L., Willey B.M. (2017). Enterobacter cloacae Complex Isolates Harboring blaNMC-A or blaIMI-Type Class A Carbapenemase Genes on Novel Chromosomal Integrative Elements and Plasmids. Antimicrob. Agents Chemother..

[40] Janecko N., Martz S.L., Avery B.P., Daignault D., Desruisseau A., Boyd D., Irwin R.J., Mulvey M.R., Reid-Smith R.J. (2016). Carbapenem-Resistant Enterobacter spp. in Retail Seafood Imported from Southeast Asia to Canada. Emerg. Infect. Dis..

[41] Brouwer M.S.M., Rapallini M., Geurts Y., Harders F., Bossers A., Mevius D.J., Wit B., Veldman K.T. (2018). Enterobacter cloacae Complex Isolated from Shrimps from Vietnam Carrying blaIMI-1 Resistant to Carbapenems but Not Cephalosporins. Antimicrob. Agents Chemother..

[42] Aubron C., Poirel L., Ash Ronald J., Nordmann P. (2005). Carbapenemase-producing Enterobacteriaceae, U.S. rivers. Emerg. Infect. Dis..

[43] Jackson C.R., Fedorka-Cray P.J., Jackson-Hall M.C., Hiott L.M. (2005). Effect of media, temperature and culture conditions on the species population and antibiotic resistance of enterococci from broiler chickens. Lett. Appl. Microbiol..

[44] Mulani M.S., Kamble E.E., Kumkar S.N., Tawre M.S., Pardesi K.R. (2019). Emerging Strategies to Combat ESKAPE Pathogens in the Era of Antimicrobial Resistance: A Review. Front. Microbiol..

[45] Tacconelli E., Carrara E., Savoldi A., Harbarth S., Mendelson M., Monnet D.L., Pulcini C., Kahlmeter G., Kluytmans J., Carmeli Y. (2018). Discovery, research, and development of new antibiotics: The WHO priority list of antibiotic-resistant bacteria and tuberculosis. Lancet Infect. Dis..

[46] Damashek J., Westrich J.R., McDonald J.M.B., Teachey M.E., Jackson C.R., Frye J.G., Lipp E.K., Capps K.A., Ottesen E.A. (2022). Non-point source fecal contamination from aging wastewater infrastructure is a primary driver of antibiotic resistance in surface waters. Water Res..

[47] (2017). Centers for Disease Control and Prevention (CDC). Outpatient Antibiotic Prescriptions—United States. https://www.cdc.gov/antibiotic-use/community/pdfs/Annual-Report-2017-H.pdf.

[48] (2018). Centers for Disease Control and Prevention (CDC). Outpatient Antibiotic Prescriptions—United States. https://www.cdc.gov/antibiotic-use/community/pdfs/Annual-Report-2018-H.pdf.

[49] Stockton B., Jones N. Antibiotics in Agriculture: The Blurred Line between Growth Promotion and Disease Prevention. https://www.thebureauinvestigates.com/stories/2018-09-19/growth-promotion-or-disease-prevention-the-loophole-in-us-antibiotic-regulations.

[50] Hanson M.L., Knapp C.W., Graham D.W. (2006). Field assessment of oxytetracycline exposure to the freshwater macrophytes Egeria densa Planch. and *Ceratophyllum demersum* L.. Environ. Pollut..

[51] Hirsch R., Ternes T., Haberer K., Kratz K.L. (1999). Occurrence of antibiotics in the aquatic environment. Sci. Total Environ..

[52] Le Page G., Gunnarsson L., Snape J., Tyler C.R. (2017). Integrating human and environmental health in antibiotic risk assessment: A critical analysis of protection goals, species sensitivity and antimicrobial resistance. Environ. Int..

[53] Suda K.J., Hicks L.A., Roberts R.M., Hunkler R.J., Taylor T.H. (2014). Trends and seasonal variation in outpatient antibiotic prescription rates in the United States, 2006 to 2010. Antimicrob. Agents Chemother..

[54] Kummerer K. (2009). Antibiotics in the aquatic environment—A review—Part I. Chemosphere.

[55] Kolpin D.W., Meyer M.T. (2002). Pharmaceuticals, hormones, and other organic wastewater contaminants in U.S. streams, 1999–2000: A National Reconnaissance. Environ. Sci. Technol..

[56] Kim S.C., Carlson K. (2007). Quantification of human and veterinary antibiotics in water and sediment using SPE/LC/MS/MS. Anal. Bioanal. Chem..

[57] Kleywegt S., Pileggi V., Yang P., Hao C., Zhao X., Rocks C., Thach S., Cheung P., Whitehead B. (2011). Pharmaceuticals, hormones and bisphenol A in untreated source and finished drinking water in Ontario, Canada—Occurrence and treatment efficiency. Sci. Total Environ..

[58] Bengtsson-Palme J., Larsson D.G. (2016). Concentrations of antibiotics predicted to select for resistant bacteria: Proposed limits for environmental regulation. Environ. Int..

[59] Graham D.W., Olivares-Rieumont S., Knapp C.W., Lima L., Werner D., Bowen E. (2011). Antibiotic resistance gene abundances associated with waste discharges to the Almendares River near Havana, Cuba. Environ. Sci. Technol..

[60] Xu J., Xu Y., Wang H., Guo C., Qiu H., He Y., Zhang Y., Li X., Meng W. (2015). Occurrence of antibiotics and antibiotic resistance genes in a sewage treatment plant and its effluent-receiving river. Chemosphere.

[61] Rodriguez-Mozaz S., Chamorro S., Marti E., Huerta B., Gros M., Sanchez-Melsio A., Borrego C.M., Barcelo D., Balcazar J.L. (2015). Occurrence of antibiotics and antibiotic resistance genes in hospital and urban wastewaters and their impact on the receiving river. Water Res..

[62] An X.L., Su J.Q., Li B., Ouyang W.Y., Zhao Y., Chen Q.L., Cui L., Chen H., Gillings M.R., Zhang T. (2018). Tracking antibiotic resistome during wastewater treatment using high throughput quantitative PCR. Environ. Int..

[63] Alexander J., Hembach N., Schwartz T. (2020). Evaluation of antibiotic resistance dissemination by wastewater treatment plant effluents with different catchment areas in Germany. Sci. Rep..

[64] Li J., Cheng W., Xu L., Strong P.J., Chen H. (2015). Antibiotic-resistant genes and antibiotic-resistant bacteria in the effluent of urban residential areas, hospitals, and a municipal wastewater treatment plant system. Environ. Sci. Pollut. Res. Int..

[65] Mao D., Yu S., Rysz M., Luo Y., Yang F., Li F., Hou J., Mu Q., Alvarez P.J. (2015). Prevalence and proliferation of antibiotic resistance genes in two municipal wastewater treatment plants. Water Res..

[66] Berendonk T.U., Manaia C.M., Merlin C., Fatta-Kassinos D., Cytryn E., Walsh F., Burgmann H., Sorum H., Norstrom M., Pons M.N. (2015). Tackling antibiotic resistance: The environmental framework. Nat. Rev. Microbiol..

[67] Sui Q., Zhang J., Tong J., Chen M., Wei Y. (2017). Seasonal variation and removal efficiency of antibiotic resistance genes during wastewater treatment of swine farms. Environ. Sci. Pollut. Res. Int..

[68] Osinska A., Korzeniewska E., Harnisz M., Felis E., Bajkacz S., Jachimowicz P., Niestepski S., Konopka I. (2020). Small-scale wastewater treatment plants as a source of the dissemination of antibiotic resistance genes in the aquatic environment. J. Hazard. Mater..

[69] Jiao Y.N., Zhou Z.C., Chen T., Wei Y.Y., Zheng J., Gao R.X., Chen H. (2018). Biomarkers of antibiotic resistance genes during seasonal changes in wastewater treatment systems. Environ. Pollut..

[70] Aydin S., Aydin M.E., Ulvi A., Kilic H. (2019). Antibiotics in hospital effluents: Occurrence, contribution to urban wastewater, removal in a wastewater treatment plant, and environmental risk assessment. Environ. Sci. Pollut. Res. Int..

[71] Zhang H., Du M., Jiang H., Zhang D., Lin L., Ye H., Zhang X. (2015). Occurrence, seasonal variation and removal efficiency of antibiotics and their metabolites in wastewater treatment plants, Jiulongjiang River Basin, South China. Environ. Sci. Process Impacts.

[72] Shen W., Chen Y., Wang N., Wan P., Peng Z., Zhao H., Wang W., Xiong L., Zhang S., Liu R. (2022). Seasonal variability of the correlation network of antibiotics, antibiotic resistance determinants, and bacteria in a wastewater treatment plant and receiving water. J. Environ. Manage.

[73] Zielinski W., Korzeniewska E., Harnisz M., Drzymala J., Felis E., Bajkacz S. (2021). Wastewater treatment plants as a reservoir of integrase and antibiotic resistance genes—An epidemiological threat to workers and environment. Environ. Int..

[74] Sabri N.A., Schmitt H., Van der Zaan B., Gerritsen H.W., Zuidema T., Rijnaarts H.H.M., Langenhoff A.A.M. (2020). Prevalence of antibiotics and antibiotic resistance genes in a wastewater effluent-receiving river in the Netherlands. J. Environ. Chem. Eng..

[75] Lekunberri I., Villagrasa M., Balcazar J.L., Borrego C.M. (2017). Contribution of bacteriophage and plasmid DNA to the mobilization of antibiotic resistance genes in a river receiving treated wastewater discharges. Sci. Total Environ..

[76] Mukherjee M., Laird E., Gentry T.J., Brooks J.P., Karthikeyan R. (2021). Increased Antimicrobial and Multidrug Resistance Downstream of Wastewater Treatment Plants in an Urban Watershed. Front. Microbiol..

[77] Manyi-Loh C., Mamphweli S., Meyer E., Okoh A. (2018). Antibiotic Use in Agriculture and Its Consequential Resistance in Environmental Sources: Potential Public Health Implications. Molecules.

[78] Capps K.A., Bateman McDonald J.M., Gaur N., Parsons R. (2020). Assessing the Socio-Environmental Risk of Onsite Wastewater Treatment Systems to Inform Management Decisions. Environ. Sci. Technol..

[79] Cho S., Hiott L.M., Barrett J.B., McMillan E.A., House S.L., Humayoun S.B., Adams E.S., Jackson C.R., Frye J.G. (2018). Prevalence and characterization of Escherichia coli isolated from the Upper Oconee Watershed in Northeast Georgia. PLoS ONE.

[80] Muller D., Greune L., Heusipp G., Karch H., Fruth A., Tschape H., Schmidt M.A. (2007). Identification of unconventional intestinal pathogenic Escherichia coli isolates expressing intermediate virulence factor profiles by using a novel single-step multiplex PCR. Appl. Environ. Microbiol..

[81] Jackson C.R., Fedorka-Cray P.J., Barrett J.B. (2004). Use of a genus- and species-specific multiplex PCR for identification of enterococci. J. Clin. Microbiol..

[82] Leader B.T., Frye J.G., Hu J., Fedorka-Cray P.J., Boyle D.S. (2009). High-throughput molecular determination of salmonella enterica serovars by use of multiplex PCR and capillary electrophoresis analysis. J. Clin. Microbiol..

[83] Clinical and Laboratory Standards Institute (CLSI) (2018). Performance Standards for Antimicrobial Susceptibility Testing.

[84] Sjolund-Karlsson M., Joyce K., Blickenstaff K., Ball T., Haro J., Medalla F.M., Fedorka-Cray P., Zhao S., Crump J.A., Whichard J.M. (2011). Antimicrobial susceptibility to azithromycin among Salmonella enterica isolates from the United States. Antimicrob. Agents Chemother..

[85] Zhao S., White D.G., McDermott P.F., Friedman S., English L., Ayers S., Meng J., Maurer J.J., Holland R., Walker R.D. (2001). Identification and expression of cephamycinase bla(CMY) genes in Escherichia coli and Salmonella isolates from food animals and ground meat. Antimicrob. Agents Chemother..

[86] Bonnet R., Recule C., Baraduc R., Chanal C., Sirot D., De Champs C., Sirot J. (2003). Effect of D240G substitution in a novel ESBL CTX-M-27. J. Antimicrob. Chemother..

[87] Fernando D.M., Tun H.M., Poole J., Patidar R., Li R., Mi R., Amarawansha G.E.A., Fernando W.G.D., Khafipour E., Farenhorst A. (2016). Detection of Antibiotic Resistance Genes in Source and Drinking Water Samples from a First Nations Community in Canada. Appl. Environ. Microbiol..

[88] Mulvey M.R., Grant J.M., Plewes K., Roscoe D., Boyd D.A. (2011). New Delhi metallo-beta-lactamase in Klebsiella pneumoniae and Escherichia coli, Canada. Emerg. Infect. Dis..

[89] Feria C., Ferreira E., Correia J.D., Goncalves J., Canica M. (2002). Patterns and mechanisms of resistance to beta-lactams and beta-lactamase inhibitors in uropathogenic Escherichia coli isolated from dogs in Portugal. J. Antimicrob. Chemother..

[90] Colom K., Perez J., Alonso R., Fernandez-Aranguiz A., Larino E., Cisterna R. (2003). Simple and reliable multiplex PCR assay for detection of blaTEM, bla(SHV) and blaOXA-1 genes in Enterobacteriaceae. FEMS Microbiol. Lett..

[91] Brinas L., Zarazaga M., Saenz Y., Ruiz-Larrea F., Torres C. (2002). Beta-lactamases in ampicillin-resistant Escherichia coli isolates from foods, humans, and healthy animals. Antimicrob. Agents Chemother..

[92] Voets G.M., Fluit A.C., Scharringa J., Cohen Stuart J., Leverstein-van Hall M.A. (2011). A set of multiplex PCRs for genotypic detection of extended-spectrum beta-lactamases, carbapenemases, plasmid-mediated AmpC beta-lactamases and OXA beta-lactamases. Int. J. Antimicrob. Agents.

[93] Yousefi S., Farajnia S., Nahaei M.R., Akhi M.T., Ghotaslou R., Soroush M.H., Naghili B., Jazani N.H. (2010). Detection of metallo-beta-lactamase-encoding genes among clinical isolates of Pseudomonas aeruginosa in northwest of Iran. Diagn. Microbiol. Infect. Dis..

[94] Dallenne C., Da Costa A., Decre D., Favier C., Arlet G. (2010). Development of a set of multiplex PCR assays for the detection of genes encoding important beta-lactamases in Enterobacteriaceae. J. Antimicrob. Chemother..

[95] R Core Team (2021). R: A Language and Environment for Statistical Computing.

[96] Stan Development Team (2021). Stan Modeling Language Users Guide and Reference Manual, 2.28. https://mc-stan.org.

[97] Gabry J., Češnovar R., Johnson A. (2022). cmdstanr: R Interface to ‘CmdStan’. https://mc-stan.org/cmdstanr/.

[98] Bürkner P.C. (2018). Advanced Bayesian Multilevel Modeling with the R Package brms. R J..

[99] Lenth R.V. (2022). Emmeans: Estimated Marginal Means, aka Least-Squares Means. R Package Version 1.8.2. https://CRAN.R-project.org/package=emmeans.

[100] Kay M. (2022). Tidybayes: Tidy Data and Geoms for Bayesian Models. R Package Version 3.0.2. http://mjskay.github.io/tidybayes/.

[101] Wiley J., Hedeker D. (2022). Brmsmargins: Bayesian Marginal Effects for ‘brms’ Models. R Package Version 0.2.0. https://CRAN.R-project.org/package=brmsmargins.

