# Supplementary material for: Distribution of Antibiotic Resistance in a Mixed-Use Watershed and the Impact of Wastewater Treatment Plants on Antibiotic Resistance in Surface Water
Source: Antibiotics (Basel). 2023 Nov 2;12(11):1586. doi: 10.3390/antibiotics12111586 (PMC10668835; doi:10.3390/antibiotics12111586)
Supplement: Supplementary file 1 [file antibiotics-12-01586-s001.zip › antibiotics-2660509-supplementary file S1.pdf]

FILE S1 A complete description of the statistical methods

Statistical analysis of data from surface water samples

Presence–absence by taxon: We fit a Bayesian logistic generalized linear mixed model (GLMM) to the presence–absence data for five different microbial taxa (AR *E. coli*, *Salmonella, Enterococcus,* ESBL-producing *Enterobacteriaceae*, and CRE) from the surface water samples. The response variable in the model was a binary variable indicating the presence of a given taxon in each surface water sample. Fixed predictors included temperature, pH, conductivity, turbidity, total *E. coli* counts, and season (categorical with four levels). Interaction terms between a taxon and the six main effects were also included. Random intercepts were fitted to each sampling site and to each combination of taxon and site, to reflect differences in baseline probability of presence among taxa. Before fitting the statistical models, the predictor variables conductivity, turbidity, and total *E. coli* counts were subjected to a log(n+1) transformation, then all continuous predictors were z-transformed to place them all on a common scale. For all fixed effects, normal prior distributions with mean 0 and standard deviation 3 were assigned. We sampled from the posterior distribution using Hamiltonian Monte Carlo (HMC), with four chains, each for 2000 warmup iterations and 1000 sampling iterations, assessing model convergence using the R-hat statistic and model fit with a posterior predictive check.

We estimated marginal trends for each continuous predictor variable separately by taxon, holding all other predictor variables constant at their mean value. We obtained a posterior distribution for the slope of each marginal trend and found the median and the 66%, 90%, 95%, and 99% quantile credible intervals (QCIs) of the distributions. For the season predictor variable, we estimated the posterior marginal mean probability of the presence for each combination of season and taxon, again calculating the median and QCIs of each marginal mean. In addition, we took contrasts (odds ratios) between the posterior mean

probabilities of presence for each pair of seasons within each taxon and calculated the median and QCIs of those ratios as well.

Presence–absence of β-lactamase-containing taxa: In addition, we fitted a separate logistic GLMM to the presence–absence data for bacterial taxa containing the β-lactamase gene. Due to sparse presence data for many of the individual taxa, we pooled all five observed taxa (*E. cloacae* complex, *E. coli, K. oxytoca, K. pneumoniae,* and *S. fonticola*) to a single presence value. We used the same six fixed effects as described in the previous model, fitting random intercepts to each sampling site. We used the same fixed-effect prior distributions, HMC sampling options, and model fit checks as above. We also generated posterior distributions of the marginal trends for each continuous predictor variable, the marginal means for each level of the categorical predictor variable season, and pairwise contrasts between the marginal means for each season. In all cases, we found the median and QCIs of the posterior distributions of the estimates.

Copy numbers of ARGs: To determine whether copy numbers of ARGs differed between surface water samples as a function of environmental conditions, we fitted a Bayesian GLMM with an absolute copy number as the response variable. Because many of the individual ARGs had relatively low copy numbers, the response variable was the sum of copy numbers across all ARGs. We included the six predictor variables listed above as fixed effects, and sampling sites were each given a random intercept. We fitted models with hurdle–gamma and hurdle–lognormal response distributions because the ARG copy number data contained many zero values and was otherwise positive and right skewed. In each model, we used the same fixed-effect prior distributions, HMC sampling options, and model fit checks as above. We fitted two different models for each of the two response distributions: one where the hurdle parameter (probability of observing exactly zero copy numbers) was allowed to vary as a function of the fixed effects, and another where the hurdle parameter was assumed to be constant across sites. We used leave-one-out cross-validation to compare among these four models. As the hurdle–gamma model with constant hurdle performed best, we present results from only that model. As above, we generated posterior distributions

of the marginal trends for each continuous predictor variable, the marginal means for each level of the categorical predictor variable season, and pairwise contrasts (ratios) between the marginal means for each season, with medians and QCIs in all cases.

Antibiotic concentration: We fitted a hurdle–gamma model identical to the one described above for copy numbers of ARGs to the antibiotic concentration data from surface water samples. As we performed for the ARG model, we summed the concentrations across all measured drugs due to the sparsity of nonzero measurements for many of the individual drugs. We estimated the same posterior marginal means and contrasts as above.

Statistical analysis of data from wastewater samples

Presence–absence by taxon: Due to the relatively low number of wastewater samples with sparse presences observed for any given taxon, as well as for β-lactamase-containing taxa as a whole, we determined that the presence–absence data were insufficient to fit a formal statistical model. We present the presence–absence data for qualitative comparisons only.

Copy numbers of ARGs: To explore the question of whether ARG copy numbers differed between wastewater treatment plants' influent and effluent, we fitted a hurdle–gamma GLMM. However, in this case, we included a separate term for each ARG instead of pooling the copy numbers across genes. We also included a fixed effect for season, a binary fixed effect for sample type (influent vs. effluent), an interaction between gene and type, and an interaction between season and type. A random intercept was fitted to each combination of season and sampling site. We assumed a constant hurdle parameter across sites. We repeated this model fit for both absolute and relative copy numbers. Fixed-effect prior distributions, HMC options, and model fit checks were as above. We estimated posterior distributions of the marginal means for each ARG separately for influent and effluent, and for influent and effluent averaged across ARGs. In addition, we estimated marginal means for each season separately for influent and effluent, averaged

across ARGs. In each case, we also calculated the posterior distribution of the copy number ratio between effluent and influent, to obtain a model prediction of the efficiency of wastewater treatment in reducing the concentration of ARGs. We further took a contrast of the efficiency ratio between pairs of seasons. As above, we calculated the median and QCIs for all the posterior distributions.

Antibiotic concentration: We fitted a model identical to the one described above for copy numbers of ARGs to the antibiotic concentration data from wastewater treatment plants' influent and effluent and estimated the same posterior marginal means and contrasts between influent and effluent.

Relative effectiveness of WWTPs: We fit an additional hurdle–gamma GLMM to the wastewater ARG absolute copy number data to compare the effectiveness of the three WWTPs in reducing the concentrations of ARGs in wastewater. This model was the same as described above but also included WWTP as an additional fixed effect, and the interaction between WWTP and sample type (influent vs. effluent). We estimated marginal means by WWTP separately for influent and effluent but averaged across all ARGs and seasons. We measured the contrast (ratio) between effluent and influent marginal means for each WWTP, then performed pairwise comparisons (also ratios) of these removal efficiency ratios for each pair of WWTPs. We fitted an identically structured model to the antibiotic concentration data and calculated the same set of marginal means and ratio contrasts.

Comparison of surface water upstream and downstream of WWTP: We additionally compared ARG copy numbers (total across all genes) and antibiotic concentrations (total across all drugs) at sampling sites upstream and downstream of the North Oconee WWTP. This was the only plant with an adequate number of both upstream and downstream sites for a quantitative comparison. We fitted another hurdle–gamma GLMM with season, location (a binary variable indicating upstream vs. downstream), and the interaction of season and location as fixed effects. Sampling sites each were assigned a random intercept. Model fitting details were as above. We estimated posterior marginal means of predicted ARG copy

numbers upstream and downstream and measured the ratio of upstream:downstream. We fitted an identical model to the total antibiotic concentration data as we performed for ARG copy numbers.

Statistical analysis was carried out using R software version 4.1.2 [95] and Stan software version 2.28 [96], including the packages cmdstanr [97], brms [98], emmeans [99], tidybayes [100], and brmsmargins [101].

References

95. R Core Team. R: A language and environment for statistical computing. R Foundation for Statistical Computing, Vienna, Austria. **2021**. https://www.R-project.org/.

96. Stan Development Team. Stan Modeling Language Users Guide and Reference Manual, 2.28. **2021**. https://mc-stan.org.

97. Gabry, J; Češnovar, R; Johnson, A. cmdstanr: R Interface to 'CmdStan'. **2022**. https://mc-stan.org/cmdstanr/; https://discourse.mc-stan.org.

98. Bürkner, P.C. Advanced Bayesian Multilevel Modeling with the R Package brms. *The R Journal* **2018**.*10*, 395-411, doi.org/10.32614/RJ-2018-017.

99. Lenth, R.V. emmeans: Estimated Marginal Means, aka Least-Squares Means. **2022**. R package version 1.8.2. https://CRAN.R-project.org/package=emmeans.

100. Kay, M. tidybayes: Tidy Data and Geoms for Bayesian Models. **2022**. doi: 10.5281/zenodo.1308151, R package version 3.0.2. http://mjskay.github.io/tidybayes/.

101. Wiley, J.; Hedeker, D. brmsmargins: Bayesian Marginal Effects for 'brms' Models. R package version 0.2.0. **2022.** https://CRAN.R-project.org/package=brmsmargins.
